# The prognostic value of DIC score combined with lactate in patients with cirrhosis complicated by variceal upper gastrointestinal bleeding: a single-center retrospective cohort study

**DOI:** 10.3389/fmed.2026.1761466

**Published:** 2026-09-09

**Authors:** Yingying Cui, Bo Liu, Nan Geng, Zhao Liu, Wen Pan, MengYun Li, Chun Lian Lai, YiXin Miao, Jingjing Wang, Haoqi Li

**Affiliations:** Emergency Department of Beijing You'an Hospital, Capital Medical University, Beijing, China

**Keywords:** DIC score, lactate, liver cirrhosis, prognosis, variceal upper gastrointestinal bleeding

## Abstract

**Objective:**

This study aimed to investigate the predictive value of the disseminated intravascular coagulation (DIC) score combined with lactate (Lac) for the prognosis of patients with cirrhosis and variceal upper gastrointestinal bleeding (VUGIB).

**Methods:**

In this retrospective cohort study, we enrolled 409 patients with liver cirrhosis and VUGIB who were admitted to Beijing You'an Hospital, Capital Medical University, between July 2023 and July 2025.The predictive outcomes were 28-day mortality, multiple organ dysfunction syndrome (MODS), and admission to the intensive care unit (ICU). Univariate baseline comparisons were performed. A multivariable logistic primary model was constructed, and two types of sensitivity analyses were established. The independence of the DIC score as a prognostic factor was assessed using Wald, likelihood-ratio, and interaction tests. Receiver operating characteristic (ROC) curves were plotted, and the areas under the curves (AUCs) of the models were compared using DeLong's test. Internal validation was conducted with 1,000 bootstrap resamples. Model calibration was evaluated using the Hosmer-Lemeshow test and calibration curves. The optimal cut-off value was determined via 10-fold cross-validation, and survival differences were analyzed using the Kaplan–Meier method. Decision curve analysis was performed to evaluate the net clinical benefit.

**Results:**

Univariate analysis revealed that lactate, DIC score, and various liver function and bleeding scores differed significantly among the three prognostic outcome groups (all *P* < 0.05). After adjusting for covariates, DIC score was identified as an independent risk factor for all three outcomes, and its predictive value was independent of Child-Pugh and MELD-Na scores, with no interaction between them. ROC analysis showed that the combined lactate + DIC model had the best discriminative performance (AUC for mortality = 0.892, for MODS = 0.889, for ICU admission = 0.851), and DeLong's test confirmed that it was superior to most conventional scores. After bootstrap correction, the model exhibited no significant optimism bias, and the Hosmer-Lemeshow test indicated good calibration. Stratification by the cross-validation-derived cutoff value revealed statistically significant differences in 28-day survival between high-and low-risk patients across all outcome groups (log-rank *P* < 0.001). Decision curve analysis demonstrated that the lactate + DIC model provided higher net clinical benefit than DIC score alone.

**Conclusion:**

The combination of DIC score and lactate independently predicts the risks of 28-day mortality, MODS, and ICU admission in patients with liver cirrhosis and VUGIB. Multidimensional validation demonstrated that this model exhibited superior discrimination, calibration, and net clinical benefit compared with any single score, and thus can be applied for early risk stratification at admission.

## 1 Introduction

Liver cirrhosis is a common chronic liver disease, categorized into compensated and decompensated stages based on disease progression (1). Decompensating events include variceal upper gastrointestinal bleeding (VUGIB), ascites, hepatic encephalopathy, and jaundice, with variceal VUGIB being the most common and dangerous complication (2). Although modern therapies for hemorrhage control have improved patient outcomes, the incidence of VUGIB remains high. Objective risk assessment tools facilitate timely risk stratification and clinical intervention for patients. The Child–Pugh score is the most widely used liver function grading tool in clinical practice, serving to evaluate the baseline hepatic reserve function. The MELD score primarily reflects the objective mortality risk in end-stage liver disease. The MELD-Na score, derived from MELD, further incorporates serum sodium, thereby enhancing the predictive performance for prognosis in cirrhotic patients with hyponatremia. Several scoring systems are used clinically to assess the prognosis of upper gastrointestinal bleeding (UGIB) patients, with the most widely used being the Rockall (RS), AIMS65, Glasgow-Blatchford (GBS), MAP, and ABC scores. The post-endoscopic Rockall (pRS) score, derived from the RS score, is more suitable for rapid emergency decision-making in liver disease patients. These scoring systems have been extensively validated in previous studies of UGIB patients (3). However, most of these studies focused on non-variceal bleeding patients, and the prognostic value of these scores is very limited for variceal bleeding patients (4). Research indicates that bleeding events in cirrhotic patients are associated with coagulation dysfunction (5). The DIC scoring system is a widely used clinical tool that provides a comprehensive assessment of a patient's coagulation status. Three major international DIC scoring systems exist: the International Society on Thrombosis and Haemostasis (ISTH) criteria, the Japanese Ministry of Health and Welfare (JMHW) criteria, and the Japanese Association for Acute Medicine (JAAM) criteria. A meta-analysis (6) suggested that the first two are useful for diagnosing the bleeding or hemorrhagic type of DIC, while the third primarily targets the organ failure or hemorrhagic type. The ISTH-DIC score can be used to assess DIC from various causes and is the most widely applied DIC scoring system internationally. It can also predict 30-day mortality in patients with liver disease (7). This study aims to explore the predictive value of DIC score combined with lactate for the prognosis of patients with cirrhosis and VUGIB.

## 2 Materials and methods

### 2.1 Study population

**Figure 1:**
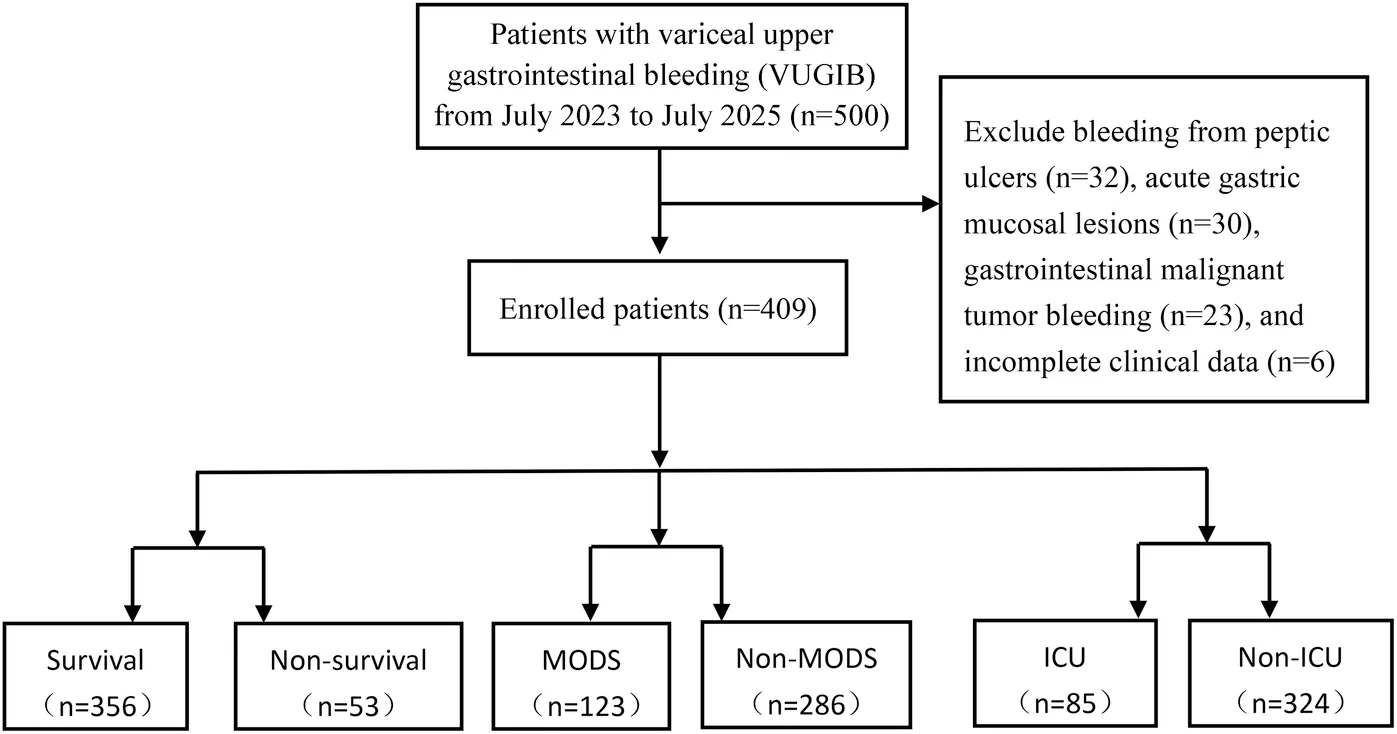
Flowchart of the study population.

Clinical data were collected from 409 hospitalized patients with cirrhosis and VUGIB admitted to our hospital between July 2023 and July 2025. Among them, 276 were male and 133 were female. The etiologies of cirrhosis included hepatitis B (183 cases), alcohol-related (74 cases), hepatitis C (30 cases), autoimmune (22 cases), and other causes (100 cases). During hospitalization, a total of 59 patients met the diagnostic criteria for overt disseminated intravascular coagulation (DIC). All patients in this cohort received vasoactive agents as part of routine care, and the medication regimen followed a unified standard. Based on the 28-day prognosis after admission, patients were divided into survival (*n* = 356) and death (*n* = 53) groups, MODS (*n* = 123) and non-MODS (*n* = 286) groups, and ICU admission (*n* = 85) and non-ICU (*n* = 324) groups. The flow chart is shown in Figure 1.

Inclusion Criteria: 1. Age ≥18 years; 2. Diagnosis of cirrhosis confirmed by ultrasound, CT, MRI, or liver biopsy; 3. Patients presented with symptoms such as hematemesis or melena and tested positive for occult blood in vomitus or stool; 4. Patients with endoscopic evidence of esophageal and/or gastric fundal variceal rupture and bleeding.; 5. Complete clinical data and laboratory examinations.

Exclusion Criteria: 1. Bleeding from ulcers; 2. Combined severe cardiac, cerebral, pulmonary, renal insufficiency, or hematologic diseases; 3. Malignant tumors; 4. Schistosomiasis infection; 5. Autoimmune diseases such as rheumatoid arthritis or systemic lupus erythematosus; 6. Use of anticoagulant or antiplatelet drugs within 3 months prior to enrollment; 7. Liver failure: specifically referring to patients with acute-on-chronic liver failure (ACLF), those who, on the basis of chronic liver disease or cirrhosis, experience acute deterioration of liver function triggered by acute insults within a short period, accompanied by progressive liver failure manifestations including severe coagulopathy, jaundice, and hepatic encephalopathy; 8. Gastrointestinal bleeding due to peptic ulcer or acute gastric mucosal lesions; 9. Pregnant or lactating women; 10. MODS had already occurred at the time of admission.

The diagnosis of MODS was based on the criteria from Prognostic scores for cirrhotic patients admitted to an intensive care unit: Which consequences for liver transplantation? and previous similar prognostic studies on bleeding in liver disease (8). Specifically, in patients with liver cirrhosis complicated by upper gastrointestinal bleeding, MODS was defined as the presence of dysfunction in two or more organs/systems, indicated by an acute increase of ≥2 points in the SOFA score within a short period (24–48 h), with the dysfunction lasting ≥24 h and not attributable to the underlying liver disease or any other single disease.

The scoring criteria and diagnostic basis for each organ dysfunction (SOFA score, 0–4 per item, with higher scores indicating more severe dysfunction) are as follows:

1. Respiratory system
  Diagnostic basis: Presence of acute respiratory distress, hypoxemia, requiring oxygen therapy or mechanical ventilation.
  Scoring criteria: PaO₂/FiO₂≥400 mmHg (0 points), 300–400 mmHg (1 point), 200–300 mmHg (2 points), 100–200 mmHg (3 points), <100 mmHg (4 points) (regardless of mechanical ventilation use).
2. Cardiovascular system
  Diagnostic basis: Presence of hypotension and signs of inadequate tissue perfusion (e.g., cold extremities, decreased urine output), requiring vasoactive agents to maintain circulation.
  Scoring criteria: Mean arterial pressure (MAP) ≥70 mmHg (0 points), MAP <70 mmHg (1 point), dopamine ≤5 or dobutamine (any dose) (2 points), dopamine >5 or epinephrine ≤0.1 or norepinephrine ≤0.1 (3 points), dopamine >15 or epinephrine >0.1 or norepinephrine >0.1 (4 points).
3. Liver
  Diagnostic basis: Acute liver injury, with elevated bilirubin and abnormal liver enzymes in the setting of underlying cirrhosis.
  Scoring criteria: Total bilirubin <20 μmol/L (0 points), 20–32 μmol/L (1 point), 33–101 μmol/L (2 points), 102–204 μmol/L (3 points), >204 μmol/L (4 points).
4. Kidney
  Diagnostic basis: Acute kidney injury, presenting with oliguria and elevated serum creatinine.
  Scoring criteria: Serum creatinine <110 μmol/L (0 points), 110–170 μmol/L (1 point), 171–299 μmol/L (2 points), 300–400 μmol/L or urine output <500 mL/day (3 points), >440 μmol/L or urine output <200 mL/day (4 points).
5. Nervous system
  Diagnostic basis: Presence of altered consciousness (lethargy, stupor, coma) after excluding other causes such as hepatic encephalopathy or cerebrovascular disease.
  Scoring criteria: Glasgow Coma Scale (GCS) score 15 (0 points), 13–14 (1 point), 10–12 (2 points), 6–9 (3 points), <6 (4 points).
6. Coagulation system
  Diagnostic basis: Acute coagulation abnormality, with thrombocytopenia and prolonged prothrombin time in the context of DIC-related parameters.
  Scoring criteria: Platelet count ≥150 × 10⁹/L (0 points), 100–150 × 10⁹/L (1 point), 50–100 × 10⁹/L (2 points), 20–50 × 10⁹/L (3 points), <20 × 10⁹/L (4 points).

### 2.2 Data collection and methods

A retrospective observational study was conducted to collect patient data, including: 1. General information: gender, age, chief complaint, estimated blood loss, etiology, heart rate, systolic blood pressure, occurrence of hepatic encephalopathy, length of hospital stay; 2. Past medical history: underlying diseases; 3. Initial laboratory tests within 24 h of admission: white blood cell count (WBC), hemoglobin (HB), platelet count (PLT), alanine aminotransferase (ALT), aspartate aminotransferase (AST), total bilirubin (TBIL), albumin (ALB), blood urea nitrogen (BUN), serum creatinine (CR), estimated glomerular filtration rate (eGFR), prothrombin time (PT), international normalized ratio (INR), D-dimer (D-D), fibrinogen (FIB), fibrinogen degradation products (FDP), and lactate (Lac), the above indicators were collected prior to all in-hospital interventions, including fluid resuscitation, endoscopic therapy, and administration of vasoactive agents; 4. Imaging and endoscopic findings: abdominal ultrasound, abdominal CT, and gastroscopy results. Patients were assessed using the Child-Pugh, MELD, MELD-Na, pRS, GBS, AIMS65, MAP, and ABC scores. The DIC score was calculated according to the International Society on Thrombosis and Haemostasis (ISTH) criteria. The ISTH-DIC scoring criteria are shown in Table 1.

**Table 1:** ISTH-DIC scoring criteria.

Parameter	Cutoff	Score
	≥100 × 10⁹/L	0
Platelet count	50–99 × 10⁹/L	1
	<50 × 10⁹/L	2
	<3 s	0
PT prolongation	3–6 s	1
	>6 s	2
Fibrinogen	≥1.0 g/L	0
	<1.0 g/L	1
	<10 mg/L	0
FDP	10–25 mg/L	2
	≥25 mg/L	3

Scoring Criteria: A score of ≥5 points is classified as overt DIC, with the score calculated once daily.

A score of <5 points suggests non-overt DIC, and the score should be recalculated within 1–2 days.

### 2.3 Observational indicators

The predictive value of individual scoring indicators, lactate alone, and their combinations for the prognosis of cirrhotic patients with VUGIB was observed and compared between groups.

### 2.4 Statistical analysis

Statistical analyses were performed using SPSS 25.0 and R 4.2. Normally distributed continuous variables were expressed as mean ± standard deviation (x¯±s) and compared between two groups using the independent-samples t-test; non-normally distributed continuous variables were presented as median (interquartile range) and compared using the Mann–Whitney U test. Categorical variables were expressed as counts (percentages) and compared using the chi-square test. Multivariable logistic regression analyses were conducted with 28-day mortality, MODS, and ICU admission as binary outcomes. The primary model included lactate, DIC score, Child-Pugh score, and ABC score. Two types of sensitivity analyses were performed: in sensitivity analysis 1, Child-Pugh was replaced by MELD-Na; in sensitivity analysis 2, ABC was sequentially replaced by pRS, GBS, AIMS65, and pRS scores to test the robustness of the DIC effect. Wald test, likelihood ratio test (LRT), and interaction terms (DIC×Child-Pugh, DIC×MELD-Na) were used to jointly verify whether the predictive value of DIC was independent of the baseline degree of liver injury. Receiver operating characteristic (ROC) curves were plotted, and the area under the curve (AUC) was used to evaluate the discriminative ability of individual indicators and the combined model; comparisons of AUCs between models were performed using DeLong's test. Internal calibration was performed using 1,000 bootstrap resamples, and the bias-corrected AUC was calculated to assess the degree of model overfitting. The Hosmer-Lemeshow (H-L) test together with calibration curves was used to evaluate model calibration. The optimal cutoff value of the model was determined using 10-fold cross-validation to avoid circular validation bias. Based on the cross-validation-derived cutoff, Kaplan–Meier survival analysis was performed, and the log-rank test was used to compare 28-day survival differences between high-and low-risk groups. Decision curve analysis (DCA) was performed to calculate the net benefit of the model at different risk thresholds, thereby evaluating its clinical utility. A two-sided *P* < 0.05 was considered statistically significant.

Missing data handling: We used listwise deletion. A total of 415 patients were initially enrolled, of whom 6 (1.45%) were excluded due to missing data on key variables, resulting in a final analytical sample size of 409 patients. Because the proportion of missing data was extremely low (<2%) and was tested to be missing completely at random (Little's MCAR test, *p* > 0.05), the impact of listwise deletion on statistical inference was negligible; therefore, more complex methods such as multiple imputation were not employed. The STROBE reporting checklist (22 mandatory items) together with the manuscript cross reference and compliance statement are provided in Supplementary Material.

## 3 Results

### 3.1 Univariate analysis

A total of 409 patients with cirrhosis and VUGIB were enrolled. In the mortality versus survival groups, age, systolic blood pressure, HB, BUN, WBC, ALT, AST, Cr, eGFR, Na⁺, Lac, PTA, INR, APTT, D-D, FDP, PT, PLT, ALB, DIC score, Child–Pugh score, Child–Pugh class, MELD score, MELD-Na score, pRS score, GBS score, AIMS65 score, MAP score, and ABC score were significantly different. Similarly, between the MODS and non-MODS groups, systolic blood pressure, HB, WBC, BUN, Cr, eGFR, K^+^, Na^+^, Lac, PTA, INR, APTT, D-D, FDP, PT, FIB, TBIL, ALB, DIC score, Child–Pugh score, Child–Pugh class, MELD score, MELD-Na score, pRS score, AIMS65 score, GBS score, MAP score, and ABC score showed significant differences. In the ICU versus non-ICU groups, heart rate, systolic blood pressure, Hb, BUN, WBC, AST, Cr, eGFR, K^+^, Na^+^, Lac, PTA, INR, APTT, D-D, FDP, PT, PLT, FIB, TBIL, ALB, DIC score, Child–Pugh score, Child–Pugh class, MELD score, MELD-Na score, pRS score, GBS score, AIMS65 score, MAP score, and ABC score were significantly different (*P* < 0.05, Table 2).

**Table 2:** Characteristics of the study cohort.

Feature	Death (*n* = 53)	Survival (*n* = 356)	t/ZX2	*P*-value	MODS (*n* = 123)	Non-MODS (*n* = 286)	t/ZX2	*P*-value	ICU (*n* = 85)	Non-ICU (*n* = 324)	t/ZX2	*P*-value
Gender (Male/Female)	42 (79%)/11 (21%)	234 (66%)/122 (34%)	3.840	0.050	91 (74%)/32 (26%)	185 (65%)/101 (35%)	3.389	0.066	62 (73%)/23 (27%)	214 (66%)/110 (34%)	1.457	0.227
Age	62 (56, 69)	59 (51, 66)	−2.312	0.021	60.13 ± 11.6	58.36 ± 11.3	1.443	0.150	61 (52,67)	59 (52,67)	−0.452	0.651
HR	90 (78, 108)	91 (79.75, 104)	-0.195	0.846	98 (80,110)	89 (79,103)	−2.391	0.170	98 (80,110)	89 (79,104)	−2.057	0.040
BP	108 (95, 124)	118 (104, 131)	−2.538	0.011	104 (91,121)	120 (109,133)	−6.577	*p* < 0.001	104 (93,121)	120 (106,133)	−5.512	*p* < 0.001
HB	64 (50, 82)	77 (61, 93)	−3.130	0.002	63 (50,79)	80 (64,99)	−6.584	*p* < 0.001	67 (53,82)	78 (61,94)	−3.474	0.001
BUN	14.56 (10.19, 25.11)	9.115 (6.6075, 12.825)	−5.493	*p* < 0.001	14.4 (9.44,22.24)	8.57 (6.31,11.29)	−8.170	*p* < 0.001	13 (8,22)	9 (6,12)	−4.876	*p* < 0.001
WBC	10 (6, 15)	5 (4, 8)	−5.742	*p* < 0.001	8 (5,12)	5 (4,7)	−5.602	*p* < 0.001	10 (6,13.5)	5 (4,7)	−7.395	*p* < 0.001
ALT	39 (16, 69)	26 (18, 35)	−3.039	0.002	25 (14,46)	27 (19,35.5)	−1.097	0.273	26 (16,59.5)	21 (18,35)	−0.236	0.813
AST	95 (35, 213)	32 (23, 52)	−5.016	*p* < 0.001	36 (23,126)	32 (23,52)	−2.206	0.027	57 (27,143)	31 (22,52)	−4.282	*p* < 0.001
CR	122 (73, 155)	61 (50.75, 77.25)	−7.349	*p* < 0.001	93 (63,142)	60 (49,73)	−8.471	*p* < 0.001	101 (63,150)	61 (50,77)	−6.589	*p* < 0.001
EGFR	55.1 (36.4, 86.4)	99.5 (85.375, 111.25)	−7.467	*p* < 0.001	70.3 (42.6,94.3)	101.7 (91.15,112.2)	−8.579	*p* < 0.001	62.4 (40.2,98.4)	99.5 (86.5,111)	−6.253	*p* < 0.001
K	4.22 (3.58, 5.25)	4.04 (3.7275, 4.4825)	−1.430	0.153	4.37 (3.8,5.19)	4 (3.7,4.41)	−4.575	*p* < 0.001	4.22 (3.76,4.95)	4.04 (3.71,4.46)	−2.398	0.016
Na	135.6 (130.1, 139.8)	138.8 (136.2, 141.22)	−3.558	*p* < 0.001	136.3 (131.7,140.2)	139.1 (136.7,141.5)	−5.150	*p* < 0.001	136.8 (130.4,140.8)	138.8 (136.2,141.3)	−2.761	0.006
Lac	4.24 (1.99, 9.52)	1.725 (1.2175, 2.59)	−6.091	*p* < 0.001	3.13 (1.62,6.37)	1.63 (1.17,2.37)	−7.643	*p* < 0.001	3.71 (1.79,7.03)	1.67 (1.19,2.5)	−7.361	*p* < 0.001
PTA	51 (35, 63)	66 (53.75, 75.25)	−4.631	*p* < 0.001	49 (35,61)	69 (59,78)	−9.326	*p* < 0.001	49 (36.5,63)	67 (56,76)	−7.032	*p* < 0.001
INR	1.62 (1.37, 2.19)	1.325 (1.21, 1.56)	−4.491	*p* < 0.001	1.63 (1.38,2.19)	1.29 (1.19,1.42)	−8.964	*p* < 0.001	1.66 (1.37,2.17)	1.31 (1.21,1.51)	−6.736	*p* < 0.001
APTT	33.1 (29.4, 43)	30.4 (27.775, 33.8)	−3.404	*p* < 0.001	32.7 (29.5,40.4)	29.9 (27.4,32.8)	−5.111	*p* < 0.001	32.4 (28.9,42.75)	30.4 (27.6,33.6)	−4.013	*p* < 0.001
DD	1,697 (951, 2,487)	434.5 (197.75, 1,067.2)	−7.187	*p* < 0.001	1,471 (624,2,487)	373 (185.5,735)	−8.483	*p* < 0.001	1,430 (564,2,695)	419 (194,965)	−6.829	*p* < 0.001
FDP	11.04 (7.34, 18.09)	2.945 (1.38, 8.0225)	−6.822	*p* < 0.001	11.82 (5.01,18.24)	2.6 (1.29,5.4)	−9.187	*p* < 0.001	10.57 (4.39,19.13)	2.84 (1.38,7.64)	−6.542	*p* < 0.001
PT	18.3 (15.4, 24.4)	15 (13.6, 17.5)	−4.610	*p* < 0.001	18.8 (15.8,24.7)	14.5 (13.2,16.2)	−9.399	*p* < 0.001	19.1 (15.45,23.9)	14.9 (13.5,17)	−7.099	*p* < 0.001
PLT	121 (66, 155)	81 (57.75, 111)	−2.939	0.003	86 (54,123)	82 (59,117)	−0.536	0.592	96 (67.5,132)	78 (55,112)	−2.390	0.017
FIB	1.91 (1.24, 2.52)	1.77 (1.43, 2.38)	−0.229	0.820	1.54 (1.05,2.27)	1.89 (1.53,2.4)	−3.739	*p* < 0.001	1.53 (1.15,2.27)	1.86 (1.47,2.4)	−3.141	0.002
TBIL	28.5 (18, 56.3)	26.15 (15.5, 47.525)	−1.093	0.275	37.1 (19.5,88.9)	24.5 (15.2,40.3)	−3.870	*p* < 0.001	29 (17.95,77.45)	25.3 (15.1,43.2)	−2.389	0.017
ALB	27.908 ± 6.3,378	29.844 ± 6.1971	−2.117	0.035	27.6 (24.2,32)	29.9 (26.35,34.55)	−3.722	*p* < 0.001	27.77 ± 7.15	30.05 ± 5.89	−3.062	0.002
DIC score	5 (4, 7)	2 (1, 3)	−8.966	*p* < 0.001	4 (3,6)	1 (1,2)	−12.318	*p* < 0.001	4 (3,6)	1 (1,3)	−9.482	*p* < 0.001
pRS score	5 (4, 6)	3 (2, 4)	−7.166	*p* < 0.001	4 (4,5)	3 (2,4)	−8.711	*p* < 0.001	4 (4,5)	3 (2,4)	−7.597	*p* < 0.001
GBS score	15 (13, 17)	13 (10, 14)	−5.836	*p* < 0.001	15 (14,16)	12 (9,13)	−11.022	*p* < 0.001	15 (13,17)	12 (10,14)	−6.986	*p* < 0.001
AIMS65 score	2 (2, 3)	1 (1, 2)	−8.163	*p* < 0.001	2 (2,3)	1 (0,2)	−11.467	*p* < 0.001	2 (2,3)	1 (1,2)	−8.335	*p* < 0.001
MAP score	5 (4, 5)	3 (3, 4)	−7.431	*p* < 0.001	5 (4,5)	3 (3,4)	−9.700	*p* < 0.001	5 (4,5)	3 (3,4)	−7.858	*p* < 0.001
ABC score	6 (5, 7)	4 (3, 5)	−7.243	*p* < 0.001	5 (4,6)	4 (3,5)	−7.693	*p* < 0.001	5 (4,7)	4 (3,5)	−5.926	*p* < 0.001
Chili-Pugh score	11 (9, 11)	8 (7, 9)	−7.320	*p* < 0.001	10 (9, 11)	8 (6, 9)	−7.627	*p* < 0.001	10 (8.5, 11)	8 (6, 9)	−7.627	*p* < 0.001
Child-Pugh class(A/B/C)	(1/18/34)	(88/190/78)	44.528	*p* < 0.001	(5/45/73)	(84/163/39)	97.990	*p* < 0.001	(3/32/50)	(86/176/62)	58.801	*p* < 0.001
MELD score	17 (11.5, 23)	11 (8, 15)	−5.196	*p* < 0.001	17 (12, 22)	10 (8, 13)	−6.513	*p* < 0.001	16 (12, 22)	11 (8, 14)	−6.513	*p* < 0.001
MELD-Na score	22 (16, 28)	13 (10,19)	−5.787	*p* < 0.001	21 (16, 27)	12 (9, 16.25)	−6.500	*p* < 0.001	20 (15, 27)	13 (10, 18)	−6.500	*p* < 0.001

### 3.2 Binary logistic regression analysis

Binary logistic regression analysis included Lac, DIC score, Child–Pugh score, and ABC score. In the model with 28-day mortality as the outcome, DIC score (OR = 1.883, 95% CI: 1.497–2.368, *P* < 0.001) and ABC score (OR = 1.947, 95% CI: 1.485–2.554, *P* < 0.001) were independent risk factors for 28-day mortality, while Lac showed a borderline significant trend (OR = 1.122, 95% CI: 0.997–1.263, *P* = 0.050). In the model with MODS as the outcome, Lac (OR = 1.187, 95% CI: 1.007–1.400, *P* = 0.042), DIC score (OR = 2.014, 95% CI: 1.627–2.494, *P* < 0.001), Child–Pugh score (OR = 1.304, 95% CI: 1.068–1.592, *P* = 0.009), and ABC score (OR = 1.670, 95% CI: 1.355–2.059, *P* < 0.001) were all independent risk factors for MODS. In the model with ICU admission as the outcome, Lac (OR = 1.247, 95% CI: 1.097–1.419, *P* = 0.001), DIC score (OR = 1.436, 95% CI: 1.205–1.711, *P* < 0.001), and ABC score (OR = 1.362, 95% CI: 1.117–1.662, *P* = 0.002) were independent risk factors for ICU admission. The results are shown in Table 3.

**Table 3:** Results of logistic regression analysis.

predictive indicator	independent variable	regression coefficient *β*	standard error	Waddle	*p*-value	OR value	95%CI
Death	Lac	0.115	0.06	3.650	0.050	1.122	0.997∼1.263
	DIC score	0.633	0.117	29.304	<0.001	1.883	1.497∼2.368
	Child-Pugh score	0.027	0.129	0.045	0.832	1.028	0.798∼1.323
	ABC score	0.667	0.138	23.246	<0.001	1.947	1.485∼2.554
MODS	Lac	0.171	0.084	4.151	0.042	1.187	1.007∼1.400
	DIC score	0.700	0.109	41.267	<0.001	2.014	1.627∼2.494
	Child-Pugh score	0.266	0.102	6.805	0.009	1.304	1.068∼1.592
	ABC score	0.513	0.107	23.078	<0.001	1.670	1.355∼2.059
ICU	Lac	0.221	0.066	11.318	0.001	1.247	1.097∼1.419
	DIC score	0.362	0.089	16.342	<0.001	1.436	1.205∼1.711
	Child-Pugh score	0.167	0.099	2.867	0.090	1.182	0.974∼1.435
	ABC score	0.309	0.102	9.274	0.002	1.362	1.117∼1.662

### 3.3 Sensitivity analysis

To exclude potential interference from the choice of liver function scores and different bleeding-related prognostic scores on the estimation of the DIC effect, two sets of sensitivity analyses were performed. (1) Sensitivity analysis 1: Child–Pugh was removed and MELD-Na was retained; a regression model was constructed incorporating Lac, DIC, and ABC, to evaluate the predictive value of DIC when only MELD-Na was used to represent baseline liver injury. (2) Sensitivity analysis 2: With Child–Pugh retained as the liver function indicator, ABC was sequentially replaced by pRS, GBS, AIMS65, and MAP, and the regression was repeated under adjustment for different bleeding risk scores. The stability of the DIC effect was tested across multiple covariate combinations to assess the robustness of the results. The results are shown in Table 4.

**Table 4:** Adjusted odds ratios for DIC score by model for three outcomes.

Model	OR for mortality	95% CI for mortality	*p*-value for mortality	OR for MODS	95% CI for MODS	*p*-value for MODS	OR for ICU	95% CI for ICU	*p*-value for ICU
M0	1.883	(1.497–2.368)	<0.001	2.014	(1.627–2.494)	<0.001	1.436	(1.205–1.711)	<0.001
M1	1.990	(1.586–2.496)	<0.001	2.027	(1.649–2.491)	<0.001	1.519	(1.284–1.798)	<0.001
M2-pRS	1.832	(1.449–2.315)	<0.001	2.002	(1.622–2.471)	<0.001	1.430	(1.194–1.713)	<0.001
M2-GBS	1.756	(1.418–2.174)	<0.001	2.464	(1.899–3.197)	<0.001	1.437	(1.206–1.712)	<0.001
M2-AIMS65	1.658	(1.343–2.046)	<0.001	1.820	(1.480–2.240)	<0.001	1.359	(1.141–1.619)	<0.001
M2-MAP	1.734	(1.403–2.144)	<0.001	2.036	(1.642–2.525)	<0.001	1.428	(1.198–1.703)	<0.001

The primary model (M0) was: Lac + DIC + Child–Pugh + ABC.

Sensitivity analysis 1 (M1) replaced Child–Pugh with MELD-Na: Lac + DIC + MELD-Na + ABC.

Sensitivity analysis 2 (M2) sequentially replaced ABC with pRS, GBS, AIMS65, or MAP, while keeping Lac, DIC, and Child–Pugh as fixed independent variables:

M2-pRS: Lac + DIC + Child–Pugh + pRS; M2-GBS: Lac + DIC + Child–Pugh + GBS; M2-AIMS65: Lac + DIC + Child–Pugh + AIMS65; M2-MAP: Lac + DIC + Child–Pugh + MAP

Across all 18 models (3 outcomes×6 models), the adjusted odds ratios for DIC score were consistently greater than 1 and statistically significant, indicating that the results were highly robust.

### 3.4 Wald test, likelihood ratio test (LRT), and interaction term analysis

To demonstrate that the predictive value of the DIC score is independent of baseline liver disease severity, we performed Wald tests, LRT, and interaction term analyses, as detailed in Tables 5–7.

**Table 5:** Wald test results for the independent effect of DIC after adjusting for baseline liver function.

Outcomes	Covariates	DIC β	SE	Wald *χ*²	df	*p*-value
Death	Child-Pugh	0.633	0.117	29.304	1	<0.001
Death	MELD-Na	0.688	0.116	35.414	1	<0.001
MODS	Child-Pugh	0.700	0.109	41.267	1	<0.001
MODS	MELD-Na	0.706	0.105	45.022	1	<0.001
ICU	Child-Pugh	0.362	0.089	16.342	1	<0.001
ICU	MELD-Na	0.418	0.086	23.745	1	<0.001

**Table 6:** Likelihood ratio test (LRT) results.

Outcomes	Nested model groups	–2LL without DIC	–2LL with DIC	LRT χ²	df	*p*-value
Death	Adjusted for Child-Pugh	212.39	178.01	34.38	1	<0.001
Death	Adjusted for MELD-Na	220.63	177.28	43.35	1	<0.001
MODS	Adjusted for Child-Pugh	325.11	270.69	54.41	1	<0.001
MODS	Adjusted for MELD-Na	327.65	266.73	60.92	1	<0.001
ICU	Adjusted for Child-Pugh	302.33	285.28	17.05	1	<0.001
ICU	Adjusted for MELD-Na	312.80	287.84	24.97	1	<0.001

**Table 7:** Results of interaction analysis (interaction coefficient β, odds ratio, and 95% CI).

Outcomes	Interaction term	β	SE	Wald χ²	df	OR	95% CI	*P*-value
Death	DIC × Child-Pugh	−0.071	0.054	1.742	1	0.931	(0.838–1.035)	0.187
Death	DIC × MELD-Na	0.001	0.013	0.008	1	1.001	(0.976–1.027)	0.930
MODS	DIC × Child-Pugh	−0.051	0.058	0.758	1	0.951	(0.848–1.066)	0.384
MODS	DIC × MELD-Na	−0.026	0.014	3.176	1	0.975	(0.948–1.003)	0.075
ICU	DIC × Child-Pugh	−0.044	0.042	1.098	1	0.957	(0.881–1.039)	0.078
ICU	DIC × MELD-Na	−0.017	0.011	2.587	1	0.983	(0.963–1.004)	0.108

Wald tests showed that, after adjusting for Child–Pugh or MELD-Na, DIC was an independent risk factor for 28-day mortality (Wald *χ*^2^ = 29.304/35.414), MODS (Wald *χ*^2^ = 41.267/45.022), and ICU admission (Wald *χ*^2^ = 16.342/23.745), all with *P* < 0.001, df=1. Likelihood ratio tests indicated that the addition of DIC significantly improved model fit (*χ*^2^=17.05–60.92, all *P* < 0.001, df=1). Interaction term analysis revealed that neither DIC × Child–Pugh nor DIC × MELD-Na was statistically significant for the outcomes of 28-day mortality, MODS, or ICU admission (all *P* > 0.05). In summary, the predictive value of DIC for adverse prognosis in patients with cirrhosis and VUGIB is independent of the baseline degree of liver injury.

### 3.5 Diagnostic performance of individual and combined indicators

**Figure 2:**
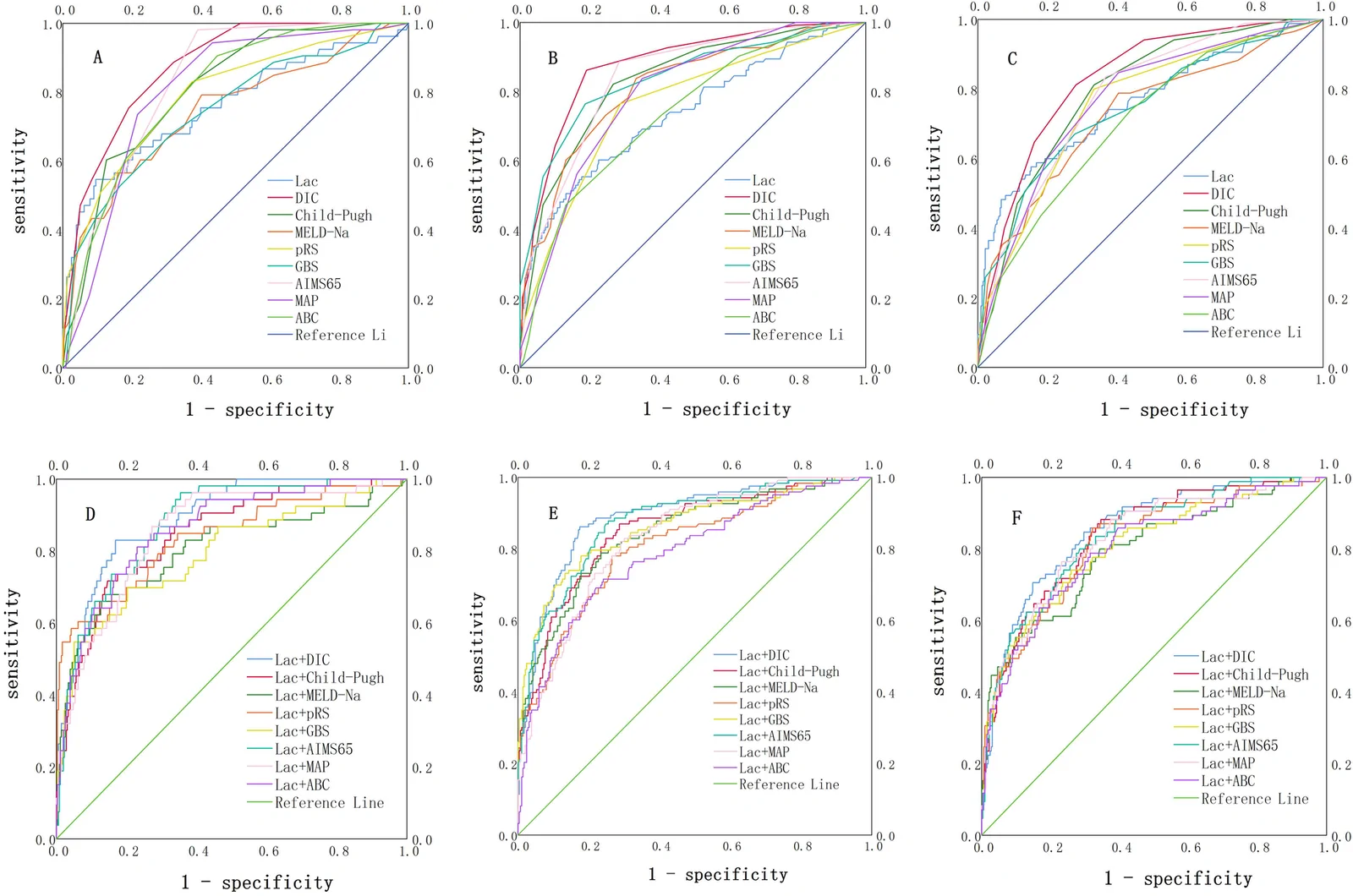
ROC curves for predicting 28 day mortality alone **(A)**, MODS alone **(B)**, ICU admission alone **(C)**, joint prediction of 28 day mortality **(D)**, joint prediction of MODS **(E)**, and joint prediction of ICU admission **(F)**.

Using lactate and each scoring system to predict the prognosis of patients with cirrhosis and variceal upper gastrointestinal bleeding (VUGIB), receiver operating characteristic (ROC) curves were plotted with sensitivity as the *Y*-axis and 1-specificity as the X-axis. Combined indicators were constructed based on logistic regression. The ROC curves for predicting 28-day mortality, development of MODS, and ICU admission are shown in Figure 2, and the corresponding AUC results are presented in Tables 8–10. Subsequent subsections describe internal validation (Section 3.5.1), AUC comparison tests (Section 3.5.2), and calibration assessment (Sections 3.5.3–3.5.4) for the combined models.

**Table 8:** Predictive value of relevant indicators for 28-day mortality in patients with cirrhosis complicated by VUGIB.

Predictive indicator	AUC	95%CI	*p*-value	Sensitivity	Specificity	Youden index	Truncation value
Lac	0.759	0.679∼0.839	<0.001	54.7%	90.4%	0.451	4.08
DIC	0.875	0.833∼0.917	<0.001	88.7%	68.0%	0.567	2.50
Child-Pugh	0.808	0.752∼0.864	<0.001	60.4%	87.4%	0.478	10.5
MELD-Na	0.746	0.668∼0.823	<0.001	56.6%	84%	0.406	21.5
pRS	0.794	0.725∼0.862	<0.001	83%	62.6%	0.456	3.50
GBS	0.747	0.671∼0.823	<0.001	67.9%	68.5%	0.364	13.50
AIMS65	0.831	0.786∼0.875	<0.001	98.1%	61.0%	0.591	1.50
MAP	0.806	0.751∼0.861	<0.001	73.6%	78.4%	0.52	4.5
ABC	0.802	0.747∼0.858	<0.001	90.6%	55.3%	0.459	4.5
Lac + DIC	0.892	0.851∼0.933	<0.001	83.0%	83.1%	0.661	0.12
Lac + Child-Pugh	0.845	0.789∼0.902	<0.001	71.7%	85.4%	0.571	0.17
Lac + MELD-Na	0.802	0.725∼0.878	<0.001	66%	87.1%	0.531	0.16
Lac + pRS	0.836	0.770∼0.903	<0.001	58.5%	95.8%	0.543	0.32
Lac + GBS	0.793	0.720∼0.867	<0.001	69.8%	79.8%	0.496	0.13
Lac + AIMS65	0.877	0.833∼0.922	<0.001	96.2%	64.6%	0.608	0.09
Lac + MAP	0.852	0.798∼0.906	<0.001	86.8%	72,8%	0.596	0.10
Lac + ABC	0.861	0.809∼0.913	<0.001	81.1%	77%	0.581	0.11

**Table 9:** Predictive value of relevant indicators for the occurrence of MODS in patients with cirrhosis complicated by VUGIB.

Predictive indicator	AUC	95%CI	*p*-value	Sensitivity	Specificity	Youden index	Truncation value
Lac	0.738	0.683∼0.793	<0.001	60.2%	77.3%	0.375	2.45
DIC	0.877	0.840∼0.914	<0.001	86.2%	80.8%	0.67	2.50
Child-Pugh	0.833	0.791∼0.876	<0.001	82.1%	73.1%	0.552	8.5
MELD-Na	0.813	0.767∼0.859	<0.001	83.7%	66.4%	0.501	14.5
pRS	0.762	0.710∼0.814	<0.001	76.4%	71%	0.474	3.50
GBS	0.841	0.797∼0.886	<0.001	76.4%	81.1%	0.575	13.50
AIMS65	0.840	0.800∼0.881	<0.001	88.6%	71.3%	0.599	1.50
MAP	0.792	0.747∼0.837	<0.001	83.7%	64.7%	0.484	3.50
ABC	0.735	0.683∼0.787	<0.001	47.2%	86.7%	0.339	5.50
Lac + DIC	0.889	0.853∼0.924	<0.001	87%	80.8%	0.678	0.07
Lac + Child-Pugh	0.853	0.812∼0.893	<0.001	86.2%	82.5%	0.687	0.07
Lac + MELD-Na	0.843	0.800∼0.886	<0.001	78.9%	76.6%	0.555	0.23
Lac + pRS	0.808	0.761∼0.855	<0.001	78.9%	72%	0.509	0.08
Lac + GBS	0.863	0.813∼0.896	<0.001	74%	83.2%	0.572	0.10
Lac + AIMS65	0.874	0.836∼0.911	<0.001	87.8%	73.8%	0.616	0.09
Lac + MAP	0.835	0.794∼0.876	<0.001	82.9%	69.6%	0.525	0.07
Lac + ABC	0.801	0.749∼0.847	<0.001	74.8%	73.4%	0.482	0.08

**Table 10:** Predictive value of relevant indicators for patients with cirrhosis complicated by VUGIB admitted to ICU.

Predictive indicator	AUC	95%CI	*p*-value	Sensitivity	Specificity	Youden index	Truncation value
Lac	0.759	0.697∼0.822	<0.001	57.6%	84.6%	0.422	3.14
DIC	0.828	0.782∼0.874	<0.001	81.2%	71.6%	0.528	2.50
Child-Pugh	0.793	0.744∼0.843	<0.001	81.2%	66.4%	0.476	8.5
MELD-Na	0.729	0.664∼0.793	<0.001	78.8%	59.3%	0.381	14.5
pRS	0.758	0.700∼0.815	<0.001	80.0%	66.4%	0.464	3.50
GBS	0.744	0.683∼0.806	<0.001	67.1%	71.9%	0.390	13.50
AIMS65	0.780	0.728∼0.831	<0.001	83.5%	63%	0.465	1.50
MAP	0.768	0.712∼0.823	<0.001	84.7%	59.3%	0.440	3.50
ABC	0.705	0.644∼0.766	<0.001	74.1%	55.6%	0.297	4.50
Lac + DIC	0.851	0.807∼0.896	<0.001	70.6%	85.8%	0.564	0.12
Lac + Child-Pugh	0.837	0.790∼0.883	<0.001	88.2%	65.4%	0.536	0.13
Lac + MELD-Na	0.794	0.736∼0.851	<0.001	80%	65.7%	0.457	0.14
Lac + pRS	0.824	0.767∼0.871	<0.001	85.9%	67.9%	0.538	0.08
Lac + GBS	0.808	0.753∼0.862	<0.001	74.1%	74.1%	0.482	0.10
Lac + AIMS65	0.839	0.789∼0.883	<0.001	80%	71.6%	0.516	0.10
Lac + MAP	0.828	0.776∼0.878	<0.001	76.5%	76.9%	0.534	0.11
Lac + ABC	0.807	0.743∼0.853	<0.001	77.6%	69.8%	0.474	0.08

The AUCs for predicting 28-day mortality were 0.759 (95% CI: 0.679–0.839) for Lac, 0.875 (95% CI: 0.833–0.917) for DIC, 0.808 (95%CI: 0.752–0.864) for Child-Pugh, 0.746 (95%CI: 0.668–0.823) for MELD-Na, 0.794 (95% CI: 0.725–0.862) for pRS, 0.747 (95% CI: 0.671–0.823) for GBS, 0.831 (95% CI: 0.786–0.875) for AIMS65, 0.806 (95% CI: 0.751–0.861) for MAP, and 0.802 (95% CI: 0.747–0.858) for ABC (all *p* < 0.001). The AUCs for the combined predictions were: Lac + DIC: 0.892 (95% CI: 0.851–0.933), Lac + Child-Pugh: 0.845, Lac + MELD-Na: 0.802, Lac + pRS: 0.836, Lac + GBS: 0.793, Lac + AIMS65: 0.877, Lac + MAP: 0.852, and Lac + ABC: 0.861 (all *p* < 0.001). The DIC score (AUC: 0.875) showed the strongest predictive ability for 28-day mortality, while the Lac + DIC combination (AUC: 0.892) demonstrated the optimal predictive performance (Figure 2, Table 8).

The AUCs for predicting MODS were 0.738 (95% CI: 0.683–0.793) for Lac, 0.877 (95% CI: 0.840–0.914) for DIC, 0.833 (95%CI: 0.791–0.876) for Child-Pugh, 0.813 (95%CI: 0.767–0.859) for MELD-Na, 0.762 (95% CI: 0.710–0.814) for pRS, 0.841 (95% CI: 0.797–0.886) for GBS, 0.840 (95% CI: 0.800–0.881) for AIMS65, 0.792 (95% CI: 0.747–0.837) for MAP, and 0.735 (95% CI: 0.683–0.787) for ABC (all *p* < 0.001). The AUCs for the combined predictions were: Lac + DIC: 0.889 (95% CI: 0.853–0.924), Lac + Child-Pugh: 0.853, Lac + MELD-Na: 0.843, Lac + pRS: 0.808, Lac + GBS: 0.863, Lac + AIMS65: 0.874, Lac + MAP: 0.835, and Lac + ABC: 0.801 (all *p* < 0.001). The DIC score (AUC: 0.877) showed the strongest predictive ability for MODS, while the Lac + DIC combination (AUC: 0.889) demonstrated the optimal predictive performance (Figure 2, Table 9).

The AUCs for predicting ICU admission were 0.759 (95% CI: 0.697–0.822) for Lac, 0.828 (95% CI: 0.782–0.874) for DIC, 0.793 (95%CI: 0.744–0.843) for Child-Pugh, 0.729 (95%CI: 0.664–0.793) for MELD-Na, 0.758 (95% CI: 0.700–0.815) for pRS, 0.744 (95% CI: 0.683–0.806) for GBS, 0.780 (95% CI: 0.728–0.831) for AIMS65, 0.768 (95% CI: 0.712–0.823) for MAP, and 0.705 (95% CI: 0.644–0.766) for ABC (all *p* < 0.001). The AUCs for the combined predictions were: Lac+DIC: 0.851(95% CI: 0.807–0.896), Lac + Child-Pugh: 0.837, Lac + MELD-Na: 0.794, Lac + pRS: 0.824, Lac + GBS: 0.808, Lac + AIMS65: 0.839, Lac + MAP: 0.828, and Lac + ABC: 0.807 (all *p* < 0.001). The DIC score (AUC: 0.828) showed the strongest predictive ability for ICU admission, while the Lac + DIC combination (AUC: 0.851) demonstrated the optimal predictive performance (Figure 2, Table 10).

#### 3.5.1 Bootstrap internal validation of joint prediction model

Prediction models constructed directly from the internal sample may suffer from optimism bias, i.e., the apparent discriminative ability of the model may be overestimated. To assess and correct for this potential bias, internal validation was performed for all 17 prediction models using the bootstrap resampling method (B=1,000 iterations). In each iteration, a subsample of equal size was drawn with replacement from the original sample, the logistic regression model was refitted, and the AUC was calculated. The process was repeated 1,000 times, and the mean of the 1,000 AUCs was taken as the bootstrap estimate. The optimism bias was calculated as bootstrap AUC−apparent AUC, and the bias-corrected AUC was defined as apparent AUC−bias. The results are shown in Table 11.

**Table 11:** Bootstrap internal validation results of each prediction model.

Predictive indicator	Outcomes	Apparent AUC	Bootstrap AUC	Bias	Corrected AUC
Lac	Death	0.759	0.761	0.002	0.757
DIC	Death	0.875	0.876	0.001	0.874
Child-Pugh	Death	0.808	0.809	0.001	0.807
MELD-Na	Death	0.746	0.747	0.001	0.745
pRS	Death	0.794	0.794	0.000	0.794
GBS	Death	0.747	0.745	−0.002	0.749
AIMS65	Death	0.831	0.831	0.000	0.831
MAP	Death	0.806	0.806	0.000	0.806
ABC	Death	0.802	0.803	0.001	0.801
Lac + DIC	Death	0.892	0.893	0.001	0.891
Lac + Child-Pugh	Death	0.845	0.846	0.001	0.844
Lac + MELD-Na	Death	0.802	0.804	0.002	0.800
Lac + pRS	Death	0.836	0.837	0.001	0.835
Lac + GBS	Death	0.793	0.796	0.003	0.790
Lac + AIMS65	Death	0.877	0.878	0.001	0.876
Lac + MAP	Death	0.852	0.853	0.001	0.851
Lac + ABC	Death	0.861	0.861	0.000	0.861
Lac	MODS	0.738	0.738	0.000	0.738
DIC	MODS	0.877	0.877	0.000	0.877
Child-Pugh	MODS	0.833	0.833	0.000	0.833
MELD-Na	MODS	0.813	0.813	0.000	0.813
pRS	MODS	0.762	0.762	0.000	0.762
GBS	MODS	0.841	0.842	0.001	0.840
AIMS65	MODS	0.840	0.841	0.001	0.839
MAP	MODS	0.792	0.793	0.001	0.791
ABC	MODS	0.735	0.736	0.001	0.734
Lac + DIC	MODS	0.889	0.889	0.000	0.889
Lac + Child-Pugh	MODS	0.853	0.853	0.000	0.853
Lac + MELD-Na	MODS	0.843	0.843	0.000	0.843
Lac + pRS	MODS	0.808	0.811	0.003	0.805
Lac + GBS	MODS	0.863	0.864	0.001	0.862
Lac + AIMS65	MODS	0.874	0.875	0.001	0.873
Lac + MAP	MODS	0.835	0.837	0.002	0.833
Lac + ABC	MODS	0.801	0.803	0.003	0.798
Lac	ICU	0.759	0.759	0.000	0.759
DIC	ICU	0.828	0.828	0.000	0.828
Child-Pugh	ICU	0.793	0.793	0.000	0.793
MELD-Na	ICU	0.729	0.727	−0.002	0.731
pRS	ICU	0.758	0.758	0.000	0.758
GBS	ICU	0.744	0.744	0.000	0.744
AIMS65	ICU	0.780	0.780	0.000	0.780
MAP	ICU	0.768	0.768	0.000	0.768
ABC	ICU	0.705	0.704	−0.001	0.706
Lac + DIC	ICU	0.851	0.852	0.001	0.850
Lac + Child-Pugh	ICU	0.837	0.836	−0.001	0.838
Lac + MELD-Na	ICU	0.794	0.794	0.000	0.794
Lac + pRS	ICU	0.824	0.825	0.001	0.823
Lac + GBS	ICU	0.808	0.809	0.001	0.807
Lac + AIMS65	ICU	0.839	0.841	0.002	0.837
Lac + MAP	ICU	0.828	0.830	0.002	0.826
Lac + ABC	ICU	0.807	0.808	0.001	0.806

Bootstrap resampling with 1,000 repetitions (B=1,000) was performed. A smaller absolute bias indicates greater model stability. The results showed that the absolute bias was≤0.003 for all models, and the bias-corrected AUCs were highly consistent with the apparent AUCs, indicating no significant optimism bias. For the core lactate+DIC model, the bias-corrected AUCs for the three outcomes were 0.891 (mortality), 0.889 (MODS), and 0.850 (ICU). This combined model demonstrated the best predictive performance across all three outcomes, with only minimal variation, confirming that its discriminative ability is stable and reliable.

#### 3.5.2 Comparison of deLong tests between combined model and single scoring model

To assess whether the superiority of the combined model over single scoring models was statistically significant, pairwise comparisons of ROC curve AUCs were performed using DeLong's nonparametric test. The DeLong test determines whether the difference in discriminative ability between two models is significant by calculating the Z statistic and *p* value. This study focused on comparing the best-performing combined model (Lac + DIC) with the best-performing single indicator (DIC), as well as the differences between Lac + DIC and other combined models. The results are shown in Table 12.

**Table 12:** Delong test comparing the AUC differences between Lac + DIC and other models.

Comparison	Outcomes	Z-value	*p*-value
Lac + DIC vs. DIC	Death	1.796	0.073
Lac + DIC vs. Lac + Child-Pugh	Death	2.025	0.043
Lac + DIC vs. Lac + MELD-Na	Death	2.872	0.004
Lac + DIC vs. Lac + GBS	Death	2.691	0.007
Lac + DIC vs. Lac + pRS	Death	1.663	0.096
Lac + DIC vs. Lac + AIMS65	Death	0.648	0.517
Lac + DIC vs. Lac + MAP	Death	1.412	0.158
Lac + DIC vs. Lac + ABC	Death	1.147	0.251
Lac + DIC vs. DIC	MODS	2.138	0.033
Lac + DIC vs. Lac + Child-Pugh	MODS	2.023	0.043
Lac + DIC vs. Lac + MELD-Na	MODS	2.098	0.036
Lac + DIC vs. Lac + pRS	MODS	2.985	0.003
Lac + DIC vs. Lac + GBS	MODS	0.938	0.348
Lac + DIC vs. Lac + AIMS65	MODS	0.706	0.480
Lac + DIC vs. Lac + MAP	MODS	2.336	0.020
Lac + DIC vs. Lac + ABC	MODS	3.397	<0.001
Lac + DIC vs. DIC	ICU	2.518	0.012
Lac + DIC vs. Lac + Child-Pugh	ICU	0.571	0.568
Lac + DIC vs. Lac + MELD-Na	ICU	2.17	0.030
Lac + DIC vs. Lac + pRS	ICU	1.128	0.259
Lac + DIC vs. Lac + GBS	ICU	1.619	0.106
Lac + DIC vs. Lac + AIMS65	ICU	0.623	0.534
Lac + DIC vs. Lac + MAP	ICU	0.941	0.347
Lac + DIC vs. Lac + ABC	ICU	1.689	0.091

A *P*-value < 0.05 indicated that the difference in AUC between the two models was statistically significant. The results showed that: (1) For 28-day mortality, Lac + DIC was significantly superior to Lac+Child-Pugh (*P*=0.043), Lac + MELD-Na (*P* = 0.004), and Lac + GBS (*P* = 0.007). (2) For MODS, Lac + DIC was significantly superior to DIC alone (*P* = 0.033), as well as to Lac + Child–Pugh (*P* = 0.043), Lac + MELD-Na (*P* = 0.036), Lac + pRS (*P* = 0.003), Lac + MAP (*P* = 0.020), and Lac + ABC (*P* < 0.001). (3) For ICU admission, Lac + DIC was significantly superior to DIC alone (*P* = 0.012) and also to Lac + MELD-Na (*P* = 0.030). Collectively, the combined Lac + DIC model demonstrated superior discriminative performance across all three adverse prognostic outcomes.

#### 3.5.3 Calibration evaluation of predictive model (Hosmer-Lemeshow test)

Model calibration refers to the agreement between predicted probabilities and observed frequencies of the outcome. In this study, the Hosmer-Lemeshow (H-L) test was used to quantitatively assess the calibration of each model: patients were divided into 10 groups based on predicted probabilities, and the observed event rate in each group was compared with the mean predicted probability from the model. The null hypothesis of the H-L test is that the calibration is good (*p* > 0.05 indicates acceptance of the null hypothesis). The results are shown in Table 13.

**Table 13:** Hosmer-Lemeshow test results for each predictive model.

Predictive indicator	Outcomes	H-L ×2	df	*P*-value
Lac	Death	4.42	8	0.817
DIC	Death	7.49	4	0.112
Child-Pugh	Death	15.14	5	0.010
MELD-Na	Death	13.42	8	0.098
pRS	Death	1.46	2	0.482
GBS	Death	19.34	7	0.007
AIMS65	Death	21.30	2	<0.001
MAP	Death	16.43	3	0.001
ABC	Death	6.05	4	0.196
Lac + DIC	Death	9.65	8	0.291
Lac + Child-Pugh	Death	4.52	8	0.808
Lac + MELD-Na	Death	10.37	8	0.240
Lac + pRS	Death	14.43	8	0.071
Lac + GBS	Death	10.26	8	0.247
Lac + AIMS65	Death	7.41	8	0.493
Lac + MAP	Death	13.00	8	0.112
Lac + ABC	Death	5.46	8	0.708
Lac	MODS	1.34	8	0.995
DIC	MODS	13.28	4	0.010
Child-Pugh	MODS	6.81	5	0.235
MELD-Na	MODS	16.06	8	0.042
pRS	MODS	5.11	2	0.078
GBS	MODS	33.09	7	<0.001
AIMS65	MODS	2.21	2	0.331
MAP	MODS	10.01	3	0.019
ABC	MODS	7.59	4	0.108
Lac + DIC	MODS	8.64	8	0.373
Lac + Child-Pugh	MODS	6.96	8	0.541
Lac + MELD-Na	MODS	10.27	8	0.247
Lac + pRS	MODS	12.01	8	0.151
Lac + GBS	MODS	20.01	8	0.010
Lac + AIMS65	MODS	4.80	8	0.779
Lac + MAP	MODS	8.50	8	0.386
Lac + ABC	MODS	4.27	8	0.832
Lac	ICU	2.13	8	0.977
DIC	ICU	9.94	4	0.041
Child-Pugh	ICU	7.45	5	0.189
MELD-Na	ICU	14.04	8	0.081
pRS	ICU	8.25	2	0.016
GBS	ICU	24.06	7	0.001
AIMS65	ICU	1.66	2	0.436
MAP	ICU	4.50	3	0.212
ABC	ICU	1.47	4	0.833
Lac + DIC	ICU	3.80	8	0.875
Lac + Child-Pugh	ICU	5.40	8	0.714
Lac + MELD-Na	ICU	9.84	8	0.276
Lac + pRS	ICU	7.67	8	0.467
Lac + GBS	ICU	4.52	8	0.807
Lac + AIMS65	ICU	15.43	8	0.051
Lac + MAP	ICU	12.83	8	0.118
Lac + ABC	ICU	4.60	8	0.799

A *P*-value > 0.05 indicated good model calibration. For the combined Lac + DIC model, the Hosmer-Lemeshow *P*-values for the three outcomes were 0.291 (mortality), 0.373 (MODS), and 0.875 (ICU), all far above 0.05, indicating excellent calibration. Some individual scores (e.g., Childp-Pugh, GBS, AIMS65, and MAP) showed poor calibration for certain outcomes, but their calibration was improved to varying degrees after combining with Lac.

#### 3.5.4 Calibration curve of the Lac + DIC combined model

**Figure 3:**
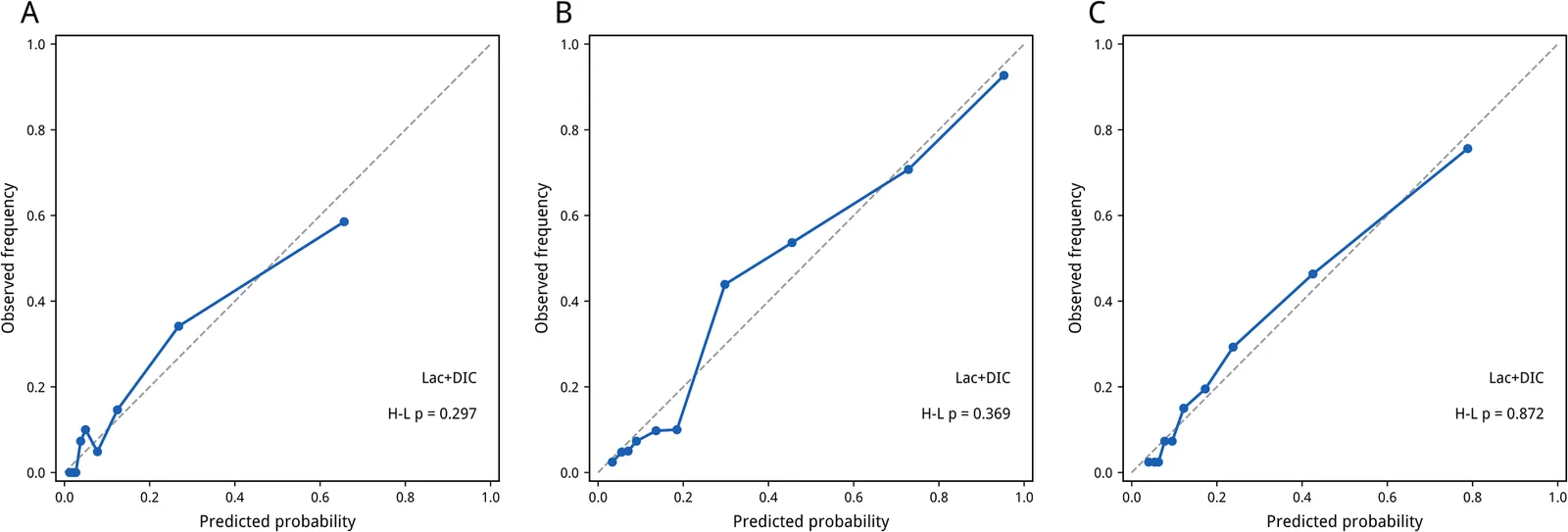
Calibration curves of the Lac + DIC combined model for predicting 28-day mortality **(A)**, MODS **(B)**, and ICU admission **(C)**.

To visually demonstrate the calibration performance of the Lac + DIC combined model, calibration curves were plotted for the three outcomes. The *X*-axis represents the model-predicted probability, the *Y*-axis represents the observed positive rate, the dashed line indicates the ideal calibration line (predicted = observed), and the solid line represents the actual calibration curve. As shown in Figure 3, the calibration curves of the Lac + DIC combined model for the three outcomes closely followed the ideal diagonal line, with good agreement in the low predicted probability range. In the high predicted probability range, the observed frequencies were slightly lower than the predicted values, but within an acceptable range. Taken together with the H-L test results (*p* > 0.05 for all three outcomes), it was confirmed that Lac + DIC possesses both good discriminative ability and good calibration.

### 3.6 28-day survival analysis

**Figure 4:**
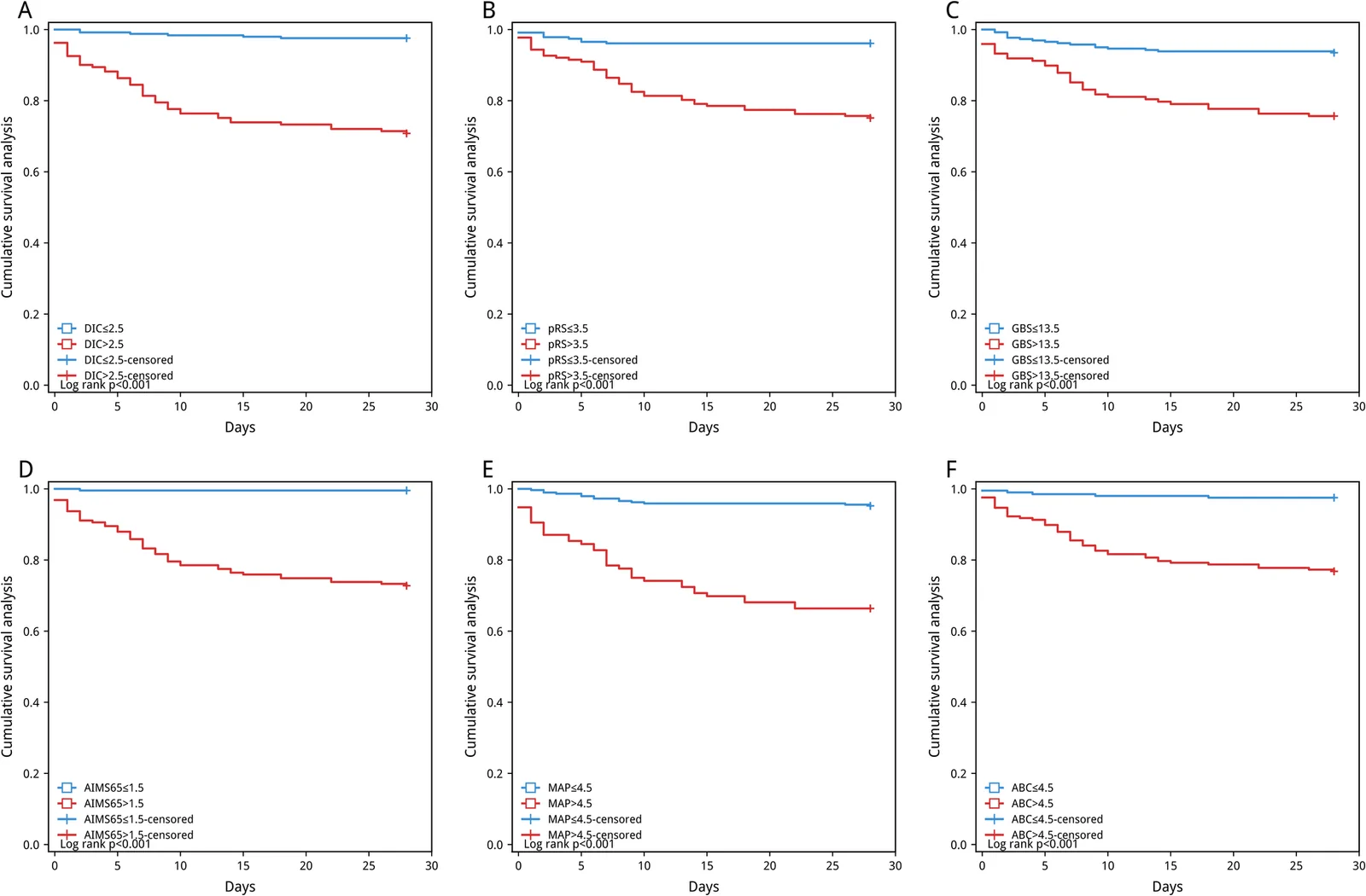
Survival curves of 28 day survival predicted by DIC **(A)**, pRS **(B)**, GBS **(C)**, AIMS65 **(D)**, MAP **(E)**, and ABC score **(F)** in patients with liver cirrhosis complicated with UGIB.

**Figure 5:**
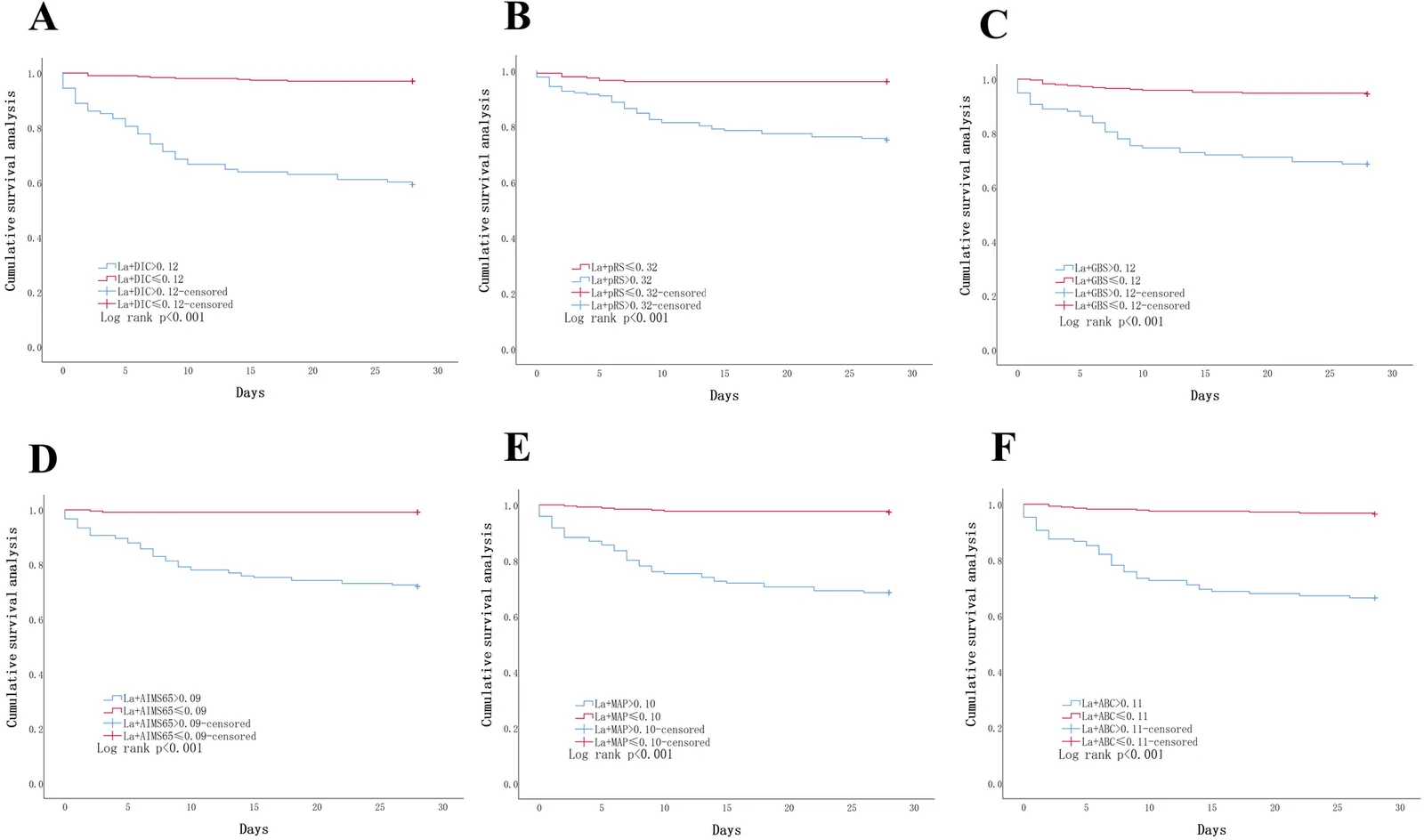
Patients with liver cirrhosis and UGIB La + DIC **(A)**, La + pRS **(B)**, La + GBS **(C)**, La + AIMS65 **(D)**, La + MAP **(E)** survival curve predicted by La + ABC score **(F)** for 28 day survival.

The 28-day survival curves for patients with cirrhosis and UGIB are shown in Figures 4, 5. To avoid circular validation bias, 10-fold cross-validation was used to determine the cut-off values (see Section 3.6.1 for details). Stratification based on the cross-validated cut-off values revealed that the differences in survival between the high-risk and low-risk groups were highly statistically significant for all indicators (all *p* < 0.001), as detailed in Table 14 and the analysis in Section 3.6.2.

**Table 14:** Comparison of youden cutoff values between the full sample and 10-fold cross-validation.

Indicator	Full sample cutoff value	CV mean	CV standard deviation	Bias
DIC	2.50	2.90	0.52	0.40
Child-Pugh	11.00	10.80	0.63	0.20
MELD-Na	22.00	20.20	2.90	1.80
pRS	3.50	3.50	0.00	0.00
GBS	13.50	14.00	0.71	0.50
AIMS65	1.50	1.50	0.00	0.00
MAP	4.50	4.10	0.52	0.40
ABC	4.50	4.60	0.32	0.10
Lac + DIC	0.123	0.124	0.004	0.001
Lac + Child-Pugh	0.172	0.168	0.011	0.004
Lac + MELD-Na	0.167	0.167	0.016	0.000
Lac + pRS	0.325	0.293	0.086	0.032
Lac + GBS	0.251	0.202	0.062	0.049
Lac + AIMS65	0.092	0.097	0.013	0.005
Lac + MAP	0.103	0.098	0.007	0.005
Lac + ABC	0.112	0.113	0.011	0.001

#### 3.6.1 Determination of cutoff values through cross-validation

Traditionally, the cut-off values for Kaplan–Meier survival analysis are determined using the Youden index on the same dataset, which carries a risk of circular validation—i.e., the cut-off determination and validation are performed on the same data, potentially artificially inflating prognostic differences between groups. Therefore, in this study, 10-fold cross-validation was used to determine the cut-off values: the sample was randomly divided into 10 parts, and in each iteration, one part was used as the validation set and the remaining nine as the training set. The optimal Youden cut-off was determined in the training set, and the final stratification threshold was taken as the mean of the 10 iterations. The results are shown in Table 14.

Bias was calculated as |mean cutoff from cross-validation-cutoff from the full sample|. The cutoff stability of the combined models was substantially superior to that of any single score: for discrete scores, the difference between the cross-validation cutoff and the full-sample cutoff was ≤ 1.8 units, whereas all lactate-containing combined models had a bias < 0.05. For Lac + DIC, the cutoff was 0.123, with a bias of only 0.001, indicating that the original cutoff was robust and reliable, and not a result of overfitting.

#### 3.6.2 Kaplan–Meier survival analysis based on cross-validation cutoff values

Kaplan–Meier survival stratification was performed based on the above cross-validated cut-off values. Detailed survival analysis results for each indicator are presented in Table 15.

**Table 15:** Kaplan–meier survival analysis results based on cross-validation truncation .

Indicator	Cutoff values	Low risk(n)	High risk(n)	28-day survival rate (high/low)	*p*-value
DIC	>2.90	248	161	0.708/0.976	<0.001
Child-Pugh	>10.80	332	77	0.584/0.937	<0.001
MELD-Na	>20.20	307	102	0.706/0.925	<0.001
pRS	>3.50	232	177	0.751/0.961	<0.001
GBS	>14.00	322	87	0.678/0.922	<0.001
AIMS65	>1.50	218	191	0.728/0.995	<0.001
MAP	>4.10	293	116	0.664/0.952	<0.001
ABC	>4.60	202	207	0.768/0.975	<0.001
Lac + DIC	>0.124	308	101	0.584/0.964	<0.001
Lac + Child-Pugh	>0.168	318	91	0.582/0.953	<0.001
Lac + MELD-Na	>0.167	329	80	0.575/0.942	<0.001
Lac + pRS	>0.293	360	49	0.367/0.939	<0.001
Lac + GBS	>0.202	349	60	0.517/0.931	<0.001
Lac + AIMS65	>0.097	251	158	0.696/0.980	<0.001
Lac + MAP	>0.098	262	147	0.687/0.973	<0.001
Lac + ABC	>0.113	285	124	0.661/0.961	<0.001

The cutoff value was the mean of the 10-fold cross-validation (CV) cutoffs. The Kaplan–Meier survival stratification *P*-values for all indicators were < 0.001, indicating that each cutoff could significantly distinguish between high-and low-risk groups. The conclusions were consistent with the original analysis, confirming that the results were not affected by circular validation bias. Survival curves are shown in Figure 4 (single scores) and Figure 5 (combined indicators).

### 3.7 Decision curve analysis (DCA)

AUC and DeLong's test only reflect the discriminative ability of a model and cannot directly answer whether the model is useful in clinical practice. Decision curve analysis (DCA) quantifies the clinical utility of a model relative to two extreme strategies (intervention for all or none) by calculating the net benefit at different threshold probabilities. The net benefit formula is: NB = TP/n – FP/n × pt/(1-pt), where pt is the threshold probability.

**Figure 6:**
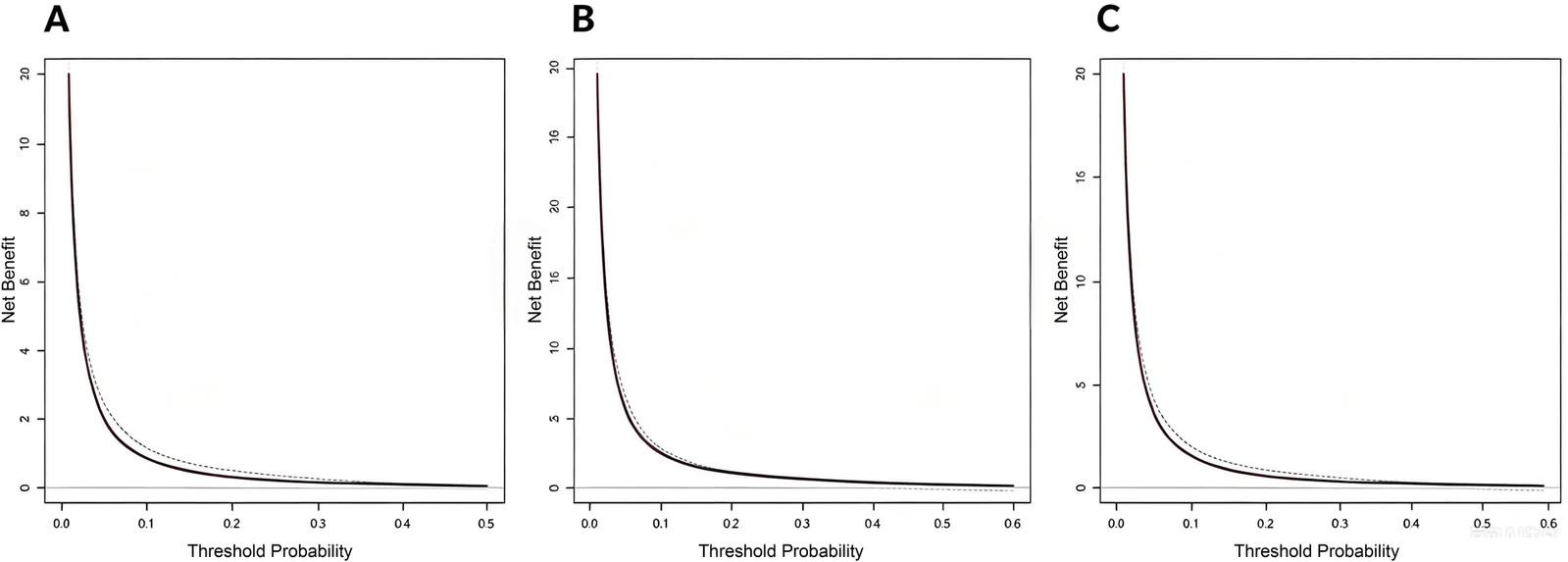
Decision curve analysis of the combined Lac + DIC model and DIC single score. **(A)** 28-day mortality; **(B)** occurrence of MODS; **(C)** ICU admission. Blue solid line, Lac + DIC combined model; red solid line, DIC single score; gray dashed line, all intervention strategies; black horizontal line, no intervention strategy.

Figure 6 presents decision curves comparing the Lac + DIC combined model and the DIC single score for the three outcomes. For predicting 28-day mortality (Figure 6A), within the threshold probability range of 0.05–0.45, the Lac + DIC combined model consistently showed a higher net benefit than the DIC single score, with a clear advantage in the clinically common range of 0.10–0.30. At a threshold of 0.10, the net benefit was 0.0864 for Lac + DIC and 0.0793 for DIC; at a threshold of 0.20, the net benefit was 0.0327 for Lac + DIC and 0.0263 for DIC. For MODS prediction (Figure 6B), the decision curves of the two models were very close, with Lac + DIC showing a slight advantage in the low-threshold region and DIC holding a slight advantage in the medium-to high-threshold region. For ICU prediction (Figure 6C), the net benefit of Lac + DIC surpassed that of DIC at thresholds >0.25, indicating greater clinical utility at higher risk thresholds. Overall, DCA confirmed that the Lac + DIC combined model has a clear clinical utility advantage in predicting 28-day mortality.

## 4 Discussion

The liver is the primary site for synthesizing most coagulation factors, maintaining systemic coagulation balance. When liver cirrhosis occurs, both coagulation factors and platelets are reduced to varying degrees (9). Consequently, cirrhotic patients are prone to bleeding at multiple sites, most commonly VUGIB (10).

A UK study reported an in-hospital mortality rate of 10% for acute VUGIB (11), which aligns with the 12.9% mortality rate in this study. VUGIB in cirrhosis is primarily attributed to esophageal or gastric varices secondary to portal hypertension. Portal hypertension leads to the formation of submucosal arteriovenous anastomoses, reducing gastric mucosal perfusion and causing ischemic and hypoxic injury (12). Worsening liver function exacerbates portal hypertension and can activate the renin-angiotensin-aldosterone system, further aggravating cirrhosis and portal hypertension, leading to dysregulated vasoconstriction/dilation and further impairment of gastric mucosal cell repair (13).

Lactate is a major product of anaerobic glycolysis, reflecting tissue hypoxia. Various critical illnesses can present with reduced tissue perfusion, leading to tissue hypoxia, increased anaerobic glycolysis, and consequent hyperlactatemia (14). Serum lactate has definite prognostic value in critically ill patients (15). In cirrhosis with VUGIB, effective blood volume drops sharply, causing tissue hypoperfusion, anaerobic metabolism, and lactate accumulation. Furthermore, impaired liver function, the primary organ for lactate metabolism, exacerbates hyperlactatemia. Thus, lactate level directly reflects the severity of shock and hepatic metabolic reserve. A prospective cohort study showed that 6-hour venous lactate clearance (VLAC), MELD-lactate (MELD-LA), and MELD-*Δ*A), and MELDactate clearance (VLAC), MELDla-day mortality in hospitalized cirrhotic patients with acute decompensation (16).Studies have shown lactate correlates with prognosis in VUGIB patients (17–19), and initial lactate on admission helps predict mortality (18). Elevated lactate is closely associated with massive bleeding in patients with VUGIB, as well as with splanchnic circulation congestion and tissue hypoxia caused by portal hypertension, and can indirectly reflect the severity of portal hypertension. Our results confirm lactate as an independent risk factor for mortality in VUGIB patients, consistent with findings by Wada et al.

The predictive value of the DIC score stems from its comprehensive assessment of coagulation system derangement. DIC results from activation of the coagulation system, leading to microvascular thrombosis and simultaneous life-threatening bleeding due to consumption of platelets and coagulation factors (20). The DIC score, incorporating PLT, fibrin-related markers (FDP or D-D), PT, and FIB, is a common tool for assessing the coagulation system, providing a more holistic view than single parameters. During hemorrhage, damaged vessel wall endothelium triggers rapid platelet activation and aggregation at the injury site (21, 22). Concurrently, coagulation factors are activated, participating with platelets in hemostasis. Conversely, disruption of the balance between platelets and coagulation factors can manifest as bleeding complications (23). D-dimer is a key marker of coagulation and fibrinolysis activation, significant for diagnosing DIC and assessing bleeding or thrombotic risk (24). FDP is a fragment produced after the degradation of plasmin, which reflects fibrinolytic function and is related to the state of the body's coagulation system. Coagulopathy in cirrhosis represents a fragile balance in a which reflects fibrinolytic function and is related to the state of the body's coagulation systemgulation factors can manifest as bleeding complications, damaged vessel wall endultiple organ failure. In patients with decompensated cirrhosis, thrombocytopenia is mainly caused by hypersplenism and reduced hepatic thrombopoietin production; prothrombin time prolongation results from impaired hepatic synthesis of coagulation factors; and elevated D-dimer/fibrin degradation products are a consequence of reduced hepatic clearance of fibrinolytic products combined with persistent low-grade fibrin turnover. These abnormalities in coagulation parameters are common in patients with advanced cirrhosis and reflect a chronic, compensatory dysregulation of hemostasis secondary to impaired liver function, rather than the pathological features of overt disseminated intravascular coagulation (DIC) as defined by the ISTH, which include systemic microthrombosis and acute progressive consumption of coagulation factors and platelets. In this study cohort, an elevated ISTH-DIC score served as a quantitative assessment of the severity of cirrhosis-related hemostatic imbalance and secondary coagulationeverrinolysis dysfunction.

In patients with cirrhosis and variceal upper gastrointestinal bleeding (VUGIB), the acute bleeding episode and hemodynamic instability impose a stress that further disrupts the already fragile rebalanced hemostatic system of cirrhosis, leading to progressive deterioration of coagulation function. The ISTH-DIC score integrates core indicators of hemostatic derangement, including platelet count, prothrombin time, and D-dimer/fibrin degradation products, thereby accurately quantifying the degree of hepatic hemostatic decompensation under acute bleeding stress. Compared with single indicators that reflect only one aspect of coagulation, the comprehensive DIC score allows systematic evaluation of the overall severity of coagulation dysfunction in cirrhotic patients under stress. Our study confirms that the DIC score, which encompasses platelet consumption, coagulation factor consumption, and hyperfibrinolysis, is tightly linked to the prognosis of cirrhotic patients with bleeding. A high DIC score may indicate more difficult-to-control bleeding, organ injury due to microvascular thrombosis, or exacerbated systemic inflammation, all of which collectively drive the risk of death and MODS. The results of the logistic regression analysis in this study confirmed that the ISTH-DIC score is an independent predictor of poor outcomes. These findings fully demonstrate that the prognostic predictive role of the DIC score is not merely an indirect reflection of overall disease severity, but independently represents the adverse prognostic risk conferred by coagulation-fibrinolysis dysfunction in these patients.

In summary, the present study confirms that the ISTH-DIC score can serve as a reliable quantitative tool for assessing the severity of hemostatic dysfunction in patients with cirrhosis and VUGIB, rather than being merely a surrogate for overall disease severity. Although previous studies (25) have demonstrated that the DIC score predicts short-term mortality in mixed cohorts of cirrhotic patients with acute decompensation, which included a considerable proportion of patients with portal hypertensive bleeding, these studies did not specifically focus on the VUGIB population, nor did they directly compare the DIC score with VUGIB-specific bleeding risk scores such as AIMS65, GBS, pRS, or ABC. The novelty of this study lies in its investigation of the prognostic value of lactate combined with the DIC score specifically in a pure VUGIB cohort, and its head-to-head comparison with existing bleeding risk scores regarding predictive performance.

A key finding of this study is the good predictive ability of the DIC score combined with lactate model for MODS and ICU admission. MODS is a leading cause of death in cirrhotic patients. Dynamic changes in coagulation parameters are closely related to the development of multiorgan failure. Our results suggest that high-risk patients can be identified early upon admission using lactate and the DIC score, providing a valuable “therapeutic window.” For predicting ICU admission, the value of the combined model lies in enabling proactive resource allocation and early intensive care intervention. When a patient presents with both high lactate and high DIC scores, it indicates not only significant bleeding and shock but also severe coagulation-inflammatory dysregulation and clinical instability. Standard ward monitoring and treatment may be insufficient to prevent deterioration, and earlier transfer to ICU for advanced life support could potentially improve outcomes.

To avoid multicollinearity, only one liver severity score (Child–Pugh) and one pre-endoscopic bleeding risk score (ABC) were included in the primary logistic regression model. Given that our study population consisted of patients with cirrhosis and VUGIB, the ABC score-which was specifically developed for patients with acute decompensation of cirrhosis and incorporates hepatic functional reserve-provides a comprehensive assessment of overall short-term mortality after bleeding. In particular, it has been shown to outperform GBS, AIMS65, MAP, and pRS in predicting 28-day mortality following bleeding. Additionally, to avoid potential interference from including multiple similar bleeding scoring systems in the same model, the ABC score was selected for inclusion in the primary logistic model.

Our results show that among individual scores, the DIC score had the best predictive ability for mortality, MODS, and ICU admission. Among combined predictors, Lac + DIC demonstrated significantly better predictive performance for all three outcomes compared to other combinations. Therefore, both the DIC score and lactate can independently predict prognosis in cirrhotic patients with VUGIB, and their combined use offers higher predictive value, consistent with findings by Rivieri and Stanley et al. (26, 27).

This study has several limitations. First, owing to the retrospective observational design and the original clinical data collection protocol, we only obtained baseline data and did not systematically collect dynamic follow-up parameters at multiple time points. Consequently, we were unable to adopt a longitudinal follow-up cohort design or apply repeated-measures or time-dependent analyses to deeply explore the temporal associations between dynamic changes in key indicators and outcome events. Future prospective studies should prespecify a protocol for repeated measurements at multiple time points and accurately record the timing of outcome events. Second, we used listwise deletion for missing data. Although the missing proportion was only 1.45%, which is extremely low, there remains a theoretical minimal risk of non-random missingness; however, such a low missing rate should have a very limited impact on the robustness of the results. Third, our cohort predominantly comprised patients with hepatitis B-related cirrhosis, with a relatively homogeneous etiological spectrum. Future multicenter prospective cohort studies with stratification by different etiologies are needed to further validate the model's performance. Fourth, we did not record the specific modalities of endoscopic therapy (e.g., ligation, sclerotherapy, or tissue adhesive injection). Endoscopic treatment modality is a potential determinant of prognosis; however, as this was a retrospective medical record analysis, and all included scores were pre-endoscopic, treatment details were not routinely documented, preventing us from assessing the potential impact of different procedures on outcomes. Future prospective studies should systematically document endoscopic treatment modalities and perform stratified analyses. Fifth, we excluded patients with ACLF to reduce the confounding effect of this severe phenotype on the coagulation-lactate-prognosis relationship, which may introduce some selection bias. Patients with ACLF often present with severe coagulation abnormalities and generally have higher DIC scores. After excluding this highcument endoscopic treatment mive ability of the DIC score for outcomes might be enhanced. If ACLF patients were included, we speculate that the overall model would retain its absolute predictive performance, but the relative discriminative ability (e.g., AUC) might decrease, and the cutoff value and weight of the DIC score within the ACLF subgroup may need separate exploration. Future studies should further validate the robustness and generalizability of our model in larger VUGIB cohorts that include ACLF patients. Sixth, this study was conducted at a single center, limiting the generalizability of our findings. Multicenter studies are warranted to confirm the rationality and scientific validity of the established model, thereby assisting clinicians in optimizing diagnostic and therapeutic strategies and reducing mortality in patients with cirrhosis and VUGIB.

## 5 Conclusion

The combination of DIC score and lac independently predicts 28-day mortality, MODS, and ICU admission in patients with cirrhosis and VUGIB.

## Data Availability

The datasets generated and analyzed for this study are not publicly available due to restrictions imposed by local ethics committee and patient-confidentiality requirements, which prevent sharing individual-level clinical data. Anonymized aggregated data may be available from the corresponding author upon reasonable written request and approval from the institutional ethics committee.
