# Supplementary material for: The prognostic value of DIC score combined with lactate in patients with cirrhosis complicated by variceal upper gastrointestinal bleeding: a single-center retrospective cohort study
Source: Front Med (Lausanne). 2026 Sep 9;13:1761466. doi: 10.3389/fmed.2026.1761466 (PMC13597319; doi:10.3389/fmed.2026.1761466)
Supplement: Supplementary file 1 [file Supplementaryfile1.pdf]

Full STROBE Checklist (22 Mandatory Items) + Manuscript Cross-Reference & Compliance Statement

Item No.	STROBE Required Content	Corresponding Manuscript Location	Compliance Explanation (Revised to Match Submitted Manuscript Text)
Title & Abstract (Item 1)	1. Title requirement: Explicitly state study design in title<br>2. Abstract requirement: Structured abstract clearly reports objective, study design, participants, methods, primary outcomes, key results, conclusions	Manuscript Title Page; Abstract section	1. The manuscript title clearly indicates a retrospective cohort design.<br>2. The structured abstract fully covers study objectives, single-center retrospective cohort enrolling 409 patients with VUGIB, construction of the Lac+DIC combined predictive model, three endpoints (28-day mortality, MODS, ICU admission), multi-dimensional verification results including ROC, KM survival analysis and DCA, as well as conclusions.
Introduction (Item 2 & Item 3)	Item 2: Describe research background, clinical gap of existing risk scores, clinical significance of this work<br>Item 3: Clearly state prespecified research objectives	Section 1 Introduction	1. This section elaborates VUGIB as the most fatal decompensation complication of cirrhosis, and clarifies that mainstream bleeding scoring systems (GBS, AIMS65, Rockall etc.) have limited predictive performance for variceal bleeding populations, and introduces ISTH-DIC and lactate's potential prognostic value.<br>2. Explicit research aim: To investigate the combined prognostic predictive value of DIC score and serum lactate in cirrhotic VUGIB patients, with three predefined adverse clinical endpoints.
Methods – Item 4 Study Design	Fully describe cohort type, recruitment time window,	Abstract opening sentence; Section	The manuscript clearly defines this as a single-center

Item No.	STROBE Required Content	Corresponding Manuscript Location	Compliance Explanation (Revised to Match Submitted Manuscript Text)
	single/multi-center, retrospective/prospective design	2.2 Data Collection and Methods	retrospective observational cohort study. Consecutive inpatients were enrolled from July 2023 to July 2025 at Beijing You'an Hospital; all clinical data were extracted retrospectively from complete electronic medical records after hospitalization.
Methods – Item 5 Setting	State hospital, department and source of enrolled patients	Section 2.1 Study Population	Study setting: Emergency Department, Beijing You'an Hospital, Capital Medical University; study population: hospitalized patients diagnosed with liver cirrhosis combined with esophagogastric variceal rupture bleeding.
Methods – Item 6 Participants	Complete inclusion & exclusion criteria; participant screening flowchart with case counts for each exclusion reason	Section 2.1 Study Population (Inclusion & Exclusion Criteria); Manuscript Figure 1	1. 5 standardized inclusion criteria and 10 detailed exclusion criteria are fully listed, explicitly excluding ACLF, peptic ulcer bleeding, malignancy, anticoagulant users and other confounding populations.<br>2. Screening workflow: Initial 415 screened patients, 6 excluded due to missing key laboratory indicators, final analytical cohort n=409; all exclusion counts and reasons are transparently recorded in the flowchart.
Methods – Item 7 Variables Definition	Clear definition of exposures, covariates, primary clinical endpoints; unified detection timing and diagnostic standards	Section 2.1 MODS diagnostic criteria; Section 2.2 Data	1. Exposure variables: admission serum lactate, ISTH-DIC score calculated via standard criteria in Table 1.<br>2. Confounding covariates:

Item No.	STROBE Required Content	Corresponding Manuscript Location	Compliance Explanation (Revised to Match Submitted Manuscript Text)
	for all indicators	Collection and Methods; Table 1 ISTH-DIC scoring criteria	Child-Pugh, MELD-Na, pRS, GBS, AIMS65, MAP, ABC scoring systems.<br>3. Primary binary endpoints: 28-day all-cause mortality, incident MODS, ICU admission after hospitalization.<br>4. All blood specimens were collected within 24 hours of admission before any fluid resuscitation, endoscopic therapy or vasoactive agent administration; MODS diagnosis strictly followed international SOFA scoring standards cited in reference literature.
Methods – Item 8 Data Sources & Measurement	Describe data extraction channels, unified laboratory testing and scoring calculation standards	Section 2.2 Data Collection and Methods	Clinical data were uniformly retrieved from hospital laboratory information system, endoscopic archives, abdominal imaging (ultrasound/CT/MRI) and inpatient medical records; all risk scores and ISTH-DIC scores were manually calculated following internationally recognized standardized formulas.
Methods – Item 9 Confounding Adjustment	Report all adjusted confounders, sensitivity analysis schemes to test result robustness	Section 2.4 Statistical Analysis; Section 3.3 Sensitivity analysis	1. Primary multivariable logistic regression adjusted for liver function (Child-Pugh) and variceal bleeding risk (ABC) scores.<br>2. Two independent sensitivity analyses were performed: Sensitivity 1 replaced Child-Pugh with MELD-Na; Sensitivity 2 sequentially

Item No.	STROBE Required Content	Corresponding Manuscript Location	Compliance Explanation (Revised to Match Submitted Manuscript Text)
			substituted ABC with pRS, GBS, AIMS65, MAP to repeatedly verify the stable independent prognostic effect of DIC score.
Methods – Item 10 Statistical Methods	Comprehensive description of all analytical tools, missing data handling, internal validation strategy, significance threshold and statistical software	Section 2.4 Statistical Analysis	1. Intergroup comparison methods: t-test, Mann-Whitney U test, chi-square test for continuous/categorical variables.<br>2. Regression tools: Multivariable logistic regression, Wald test, likelihood ratio test, interaction term analysis.<br>3. Model validation tools: ROC & DeLong test, 1000-time Bootstrap internal validation, Hosmer-Lemeshow calibration test, calibration curves, 10-fold cross-validation for optimal cutoff, Kaplan-Meier Log-rank survival analysis, decision curve analysis (DCA).<br>4. Missing data: Listwise deletion adopted; Little's MCAR test confirmed data were completely randomly missing (missing rate=1.45%).<br>5. Software: SPSS 25.0, R 4.2; two-sided P < 0.05 defined as statistical significance.
Results – Item 11 Baseline Characteristics	Report total sample size, demographic and clinical baseline data of all outcome subgroups, missing data proportion	Section 3.1 Univariate Analysis; Table 2; Missing data paragraph in 2.4	1. Final analytical sample: 409 patients (276 male, 133 female), grouped into three paired binary cohorts: death/survival, MODS/non-MODS,

Item No.	STROBE Required Content	Corresponding Manuscript Location	Compliance Explanation (Revised to Match Submitted Manuscript Text)
			ICU/non-ICU.2. Full baseline clinical, laboratory and scoring indicators of each subgroup were quantified and displayed in Table 2, with all median/mean values and test statistics provided.3. Missing data proportion clearly reported as 1.45%.
Results – Item 12 Exposure & Covariate Baseline Differences	Present univariate intergroup differences of lactate, DIC score and all adjusted risk scores	Section 3.1 Univariate Analysis; Table 2	All lactate, coagulation indicators, liver function scores and bleeding risk scores showed statistically significant differences across three adverse outcome subgroups (all $P < 0.05$), complete statistical values listed in Table 2.
Results – Item 13 Outcome Event Counts & Incidence	Report absolute number and incidence rate of each predefined primary endpoint	Section 2.1 Study Population; Section 3.1	28-day mortality: 53 cases (12.9%); MODS occurrence: 123 cases; ICU admission: 85 cases; all event counts were clearly stratified by comparison groups.
Results – Item 14 Effect Size Estimation	Report adjusted OR, 95% CI, P values from multivariable regression; results of sensitivity, Wald, LRT and interaction tests	Section 3.2 Binary Logistic Regression (Table 3); Section 3.3 Sensitivity Analysis (Table 4); Section 3.4 Wald / LRT / Interaction Test (Table 5–7)	After adjusting multiple confounders, DIC score was confirmed as an independent risk factor for all three endpoints (all $OR > 1$, $P < 0.001$). All interaction terms between DIC and liver scores had $P > 0.05$, verifying its independent predictive value unaffected by baseline hepatic injury severity.
Results – Item 15 Model Discrimination & Calibration	Report ROC-AUC, Bootstrap corrected AUC, Hosmer-Lemeshow P-value and calibration	Section 3.5 Single & Combined Diagnostic	The Lac+DIC combined model achieved the highest AUC among all single and combined predictors for all three

Item No.	STROBE Required Content	Corresponding Manuscript Location	Compliance Explanation (Revised to Match Submitted Manuscript Text)
	curves	Performance; Table 8–11, Table 13; Figure 3	outcomes. Bootstrap correction showed negligible optimism bias; H-L test P-values of Lac+DIC were all >0.05, and calibration curves closely matched the ideal reference line, proving excellent discrimination and calibration simultaneously.
Results – Item 16 Stratified Survival Outcomes	Cross-validated optimal cutoff values, Kaplan-Meier curves and Log-rank P-values	Section 3.6 28-Day Survival Analysis; Table 14, Table 15; Figure 4 & Figure 5	Cutoff thresholds were calculated via 10-fold cross-validation to avoid circular validation bias. All high-risk vs low-risk subgroups had significant 28-day survival differences (all Log-rank $P < 0.001$), visualized in survival figures.
Results – Item 17 Net Clinical Benefit Analysis	Report decision curve analysis comparing clinical utility of single vs combined model	Section 3.7 Decision Curve Analysis; Figure 6	DCA curves demonstrated that the Lac+DIC combined model yielded higher net clinical benefit than DIC score alone within most clinically relevant risk thresholds, especially for predicting 28-day mortality.
Discussion – Item 18 Core Result Interpretation	Interpret key findings aligned with research objectives; elaborate pathophysiological mechanisms of lactate and DIC as prognostic biomarkers	Section 4 Discussion Paragraphs 1–4	Interpret predictive mechanisms from two dimensions: 1) Lactate reflects hemorrhagic shock-induced tissue hypoperfusion and impaired hepatic lactate clearance; 2) ISTH-DIC score comprehensively quantifies disrupted hemostatic rebalance in cirrhotic patients after acute variceal bleeding, linking coagulation-fibrinolysis dysfunction to multi-organ

Item No.	STROBE Required Content	Corresponding Manuscript Location	Compliance Explanation (Revised to Match Submitted Manuscript Text)
			injury and death risk.
Discussion – Item 19 Comparison with Published Literature	Compare this model's advantages with existing scoring systems, clarify study novelty	Section 4 Discussion Paragraph 5	Prior cohort studies of DIC score mixed all acute decompensation cirrhosis patients without exclusive VUGIB stratification, and lacked head-to-head comparison with multiple variceal bleeding-specific risk scores. This study innovatively validated the superior performance of Lac+DIC within a pure VUGIB single-center cohort.
Discussion – Item 20 Study Limitations	Objectively and comprehensively list all design limitations and potential bias sources	Final paragraph of Section 4 Discussion	Six clear limitations were fully elaborated:
Item 21 Research conclusions	Draw objective conclusions strictly based on current study data without overstating causal inference or extrapolating beyond study scope	Abstract Conclusions; Final paragraph of Discussion	The combination of DIC score and serum lactate can independently predict 28-day mortality, MODS and ICU admission risk upon admission in cirrhotic VUGIB patients. The combined model exhibits superior discrimination, calibration and net clinical benefit compared with traditional single risk scores, and supports early in-hospital risk stratification in clinical practice.
Other Essential Information (Item 22)	Item 22: Ethical approval, informed consent, funding disclosure, conflict of interest statement and standardized references	End of Inclusion criteria; Reference list at manuscript end	1. Ethics statement (or method section):<br>The study protocol was retrospective, and therefore written informed consent was

Item No.	STROBE Required Content	Corresponding Manuscript Location	Compliance Explanation (Revised to Match Submitted Manuscript Text)
			waived.<br>2. References/Literature search :<br>References should include high-quality, authoritative publications from the past 5 years, focusing on hepatology, emergency medicine, and coagulation disorders, and should be formatted in accordance with the journal's uniform requirements for references.<br>3.Conflict of interest statement: All authors declare that they have no conflicts of interest.
